# Six New Species of Genus *Pedetontus* Silvestri, 1911 (Microcoryphia: Machilidae), from Southern China †

**DOI:** 10.3390/insects16090916

**Published:** 2025-09-01

**Authors:** Chen-Yang Shen, Ting Yang, Jie-Hong Ji, Jia-Yong Zhang

**Affiliations:** 1 College of Life Sciences, Zhejiang Normal University, Jinhua 321004, China; 2 Key Laboratory of Wildlife Biotechnology, Conservation and Utilization of Zhejiang Province, Zhejiang Normal University, Jinhua 321004, China

**Keywords:** Microcoryphia, Archaeognatha, *Pedetontus*, China, taxonomy, morphology, *COX1*

## Abstract

Bristletails (Microcoryphia) represent a relatively understudied group of class Insecta. These wingless hexapods, which retain numerous plesiomorphic structures, remain less well understood compared to most other insect lineages. Through detailed morphological examination of specimens collected from southern China, we describe six new species (*Pedetontus* (*Verhoeffilis*) *elegans* **sp. n.** from Zhejiang; *P.* (*V.*) *hezhouensis* **sp. n.**, *P.* (*V.*) *jinxiuensis* **sp. n.,** and *P.* (*V.*) *nanningensis* **sp. n.** from Guangxi; and *P.* (*V.*) *shenzhenensis* **sp. n.** and *P.* (*V.*) *xanthospilus* **sp. n.** from Guangdong) of subgenus *Verhoeffilis* Paclt, 1972. This subgenus is distinguished from other members of the Petrobiinae by several diagnostic features: paired ocelli shoe-shaped (fusiform-shaped) and submedian; two pairs of retractile vesicles on abdominal segments II–V; parameres restricted to coxite IX; penis opening small and apical; male genitalia not exceeding coxite IX; and ovipositor of primary type. The new species can be distinguished from known species and each other by the morphology of the compound eyes, maxilla, labial palps, legs, and genitalia; here, we provide detailed illustrations, tables, and descriptions. Furthermore, we sequence the *COX1* gene to construct a phylogenetic tree of Microcoryphia. This contribution increases the total number of reported *Pedetontus* species in China to seventeen.

## Simple Summary

Bristletails (Microcoryphia), due to their remote habitats and cryptic lifestyles, have long remained among the least studied insect groups. Research on these insects, which retain numerous ancestral morphological traits, is of significant importance for understanding the early origins and evolutionary pathways of insects. Through the examination of specimens collected in southern China, we identified six previously undescribed species within the genus *Pedetontus* Silvestri, 1911. Utilizing dissections and high-resolution imaging under a stereomicroscope, this study provides comprehensive color documentation of various morphological structures, greatly surpassing the quality and detail of earlier records, which were largely confined to monochromatic line drawings and incomplete color images.

## 1. Introduction

Insects represent the most widespread and adaptable group of animals on Earth, primarily due to their well-developed wings and diverse modes of metamorphosis. However, bristletails (Microcoryphia, also referred to as Archaeognatha) differ significantly from the majority of insects: they are wingless and thus unable to fly, their juveniles and adults differ only in body size and sexual maturity, and they molt throughout their entire lives [1,2]. With cryptic habitats and lifestyles, bristletails retain numerous plesiomorphic characteristics of ancestral insects, such as monocondylous mandibles, abdominal appendages not fully reduced on each segment, and well-developed caudal filaments and cerci. As the sister group to all other extant insect lineages, bristletails hold significant value in understanding the evolutionary origins of insects [3,4]. Nevertheless, knowledge regarding bristletails remains limited compared to that of other insect groups. Many species have yet to be discovered, and numerous regions remain taxonomically unexplored or poorly understood, often described as “black holes” in entomological research [5].

The genus *Pedetontus* Silvestri, 1911, is classified under the family Machilidae, subfamily Petrobiinae, and has a distribution extending along North America, the Pacific coast of North Asia, East Asia, and Southeast Asia [6,7]. The genus can be distinguished from other genera based on the following characteristics: antennae scaled except for the flagellum; shoe-shaped (fusiform or dumbbell-shaped) ocelli located subinferior to the compound eyes; mandibles with four typical apical teeth; maxillary palps and all thoracic legs scaled; fore femora not swollen; meso- and metacoxae bearing styli; abdominal segments II–V or VI possessing two pairs of retractive vesicles; penis shorter than the coxite IX; parameres on coxite IX, coxite XIII without parameres; and ovipositor primary type, without fossil claw.

*Pedetontus* was originally established as a subgenus of *Petrobius* Leach, 1809, by the distinguished Italian entomologist Filippo Silvestri in 1911, who described 18 new species [8,9,10,11,12,13]. Subsequently, in 1936, Silvestri treated *Pedetontus* as a genus in his paper “Descripzione di alcuni Machilidae (Thysanura) della Cina”. In 1972, *Pedetontus* was subdivided by Paclt into two subgenera: *Pedetontus sensu stricto* Silvestri, 1911 (=*Pedetontus s. str.* = *Pedetontus* (*P.*)), and *Verhoeffilis* Paclt, 1972. Subgenus *Pedetontus Verhoeffilis* is characterized by the presence of two pairs of retractile vesicles restricted to coxites II–V and posterior angles of abdominal sternites ≤90°. In contrast, the subgenus *Pedetontus s. str.* possesses retractile vesicles on coxites II–VI and exhibits obtuse posterior angles of sternites [14]. Since 1960, an additional 17 species have formally been described by subsequent taxonomists [6,14,15,16,17,18,19,20,21,22,23,24,25,26]. The three Chinese *Pedetontus* species—*P.* (*Verhoeffilis*) *savioi*, *P.* (*V.*) *bianchii*, and *P.* (*V.*) *fukiensis*—were described by Silvestri [12]. In 1943, Silvestri described three more species: *P.* (*V.*) *formosanus*, *P.* (*V.*) *issikii*, and *P.* (*V.*) *sauterii* [13]. In 1965, the Japanese scholar Uchida described a new species, *P.* (*V.*) *uariensis*, based on specimens collected in Taiwan, China, by the Japan Lepidoptera Society [22]. In 1991, Xue & Yin described *P.* (*V.*) *zhejiangensis* from Tianmu Mountain [24]. In 2008, based on specimens collected in Beijing, Zhang reported the complete mitochondrial genome sequence of *P.* (*V.*) *silvestrii*, the type locality of which is Korea [19,26]. Two years later, the new species *P.* (*V.*) *hainanensis* and *P.* (*V.*) *zhoui* were described from Hainan and Fujian, respectively [25]. To date, a total of 11 species of *Pedetontus* have been documented in China, along with a cumulative count of 35 extant species of bristletails reported nationwide [9,12,13,22,24,25,27,28,29,30,31,32,33,34,35,36,37,38,39,40,41,42].

Southern China is characterized by complex terrain, diverse climatic conditions, and abundant biological resources; however, research on bristletail groups remains comparatively limited. Through the dissection and morphological examination of bristletail specimens collected in southern China, this study identifies and formally describes six new species of *Pedetontus* and presents the first comprehensive set of detailed color photographs for the species within this genus.

## 2. Materials and Methods

### 2.1. Specimen Collection and Morphological Examination

After capture, some specimens were immediately preserved in 70% ethanol for subsequent dissection and morphological examination, while the remaining individuals were reared on a diet of moss and algae. Following molting and the complete development of scales, whole-body images were captured. Specimens were dissected and observed using an Olympus SZX16 stereomicroscope (Olympus Corporation, Tokyo, Japan) and a Novel N-117M microscope (Novel Corporation, Ningbo, China). Morphological structures were mounted on a temporary slide and imaged with an Olympus DP73 camera (Olympus Corporation, Tokyo, Japan). The resulting images were post-processed and assembled into figure plates using Affinity Photo 2 (Serif Ltd., Nottingham, UK, available from: https://affinity.serif.com/zh-cn/ (accessed on 12 December 2023)).

### 2.2. DNA Extraction, PCR, DNA Sequencing, and Phylogenetic Tree

The muscle tissue dissected and isolated from the examined specimens was utilized for DNA extraction. Total genomic DNA extraction was conducted using the Ezup Column Animal Genomic DNA Purification Kit (Sangon Biotech Company, Shanghai, China), following the manufacturer’s standard operating procedures.

This study utilized Sanger sequencing to obtain *COX1* gene sequences. Following DNA extraction, the *COX1* gene was amplified using universal primers LCO1490 (5′-GGTCAACAAATCATAAAGATATTGG-3′) and HCO2198 (5′-TAAACTTCAGGGTGACCAAAAATCA-3′) [43]. Eleven PCR products were submitted to Zhejiang Youkang Biotechnology Co., Ltd. (Wenzhou, China) for Sanger sequencing. The concatenated maximum matrix was subjected to best-fit model selection through PartitionFinder 2.2.1 [44] in Python 2.7, which identified “GTR+I+G” as the optimal evolutionary model.

Phylogenetic construction was performed using 41 *COX1* sequences, including 11 newly sequenced *COX1* genes from this study and 30 Microcoryphia *COX1* sequences (comprising Meinertellidae and Machilidae) downloaded from NCBI [27,36,42,45,46,47,48,49,50,51]. *Nesomachilis australica* (AY793551) from Meinertellidae served as the outgroup [52]. Detailed *COX1* accession numbers are provided in Table S1. A Bayesian inference (BI) tree was constructed using MrBayes 3.2 [53] with 10 million generations of Markov chain Monte Carlo (MCMC) simulations, sampling every 1000 generations (25% initial samples discarded as burn-in). The final phylogenetic tree was visualized and refined using ChiPlot [54] (https://www.chiplot.online, accessed on 26 June 2025).

## 3. Results

### 3.1. Taxonomy

Family ***Machilidae*** Lubbock, 1873Subfamily ***Petrobiinae*** Kaplin 1985Genus ***Pedetontus*** Silvestri, 1911Subgenus ***Pedetontus*** (***Verhoeffilis***) Paclt, 1972Type species: ***Petrobius*** (***Pedetontus***) ***calcaratus*** (Silvestri, 1911)

**Subgeneric diagnosis.** Flagellum of antenna devoid of scales, maxilla and legs possess scales. Paired ocelli shoe-shaped (dumbbell-shaped), submedian, relatively close to each other. Mandibles typical, with four apical teeth. Thorax normal. Coxal stylets on midlegs and hindlegs. Forelegs of male not swollen, without sensory fields. Two pairs of retractile vesicles on the abdominal segments II–V. Paramera restricted to the IXth. Penis opening little, apical. Male genitalia not exceeding IXth coxites. Ovipositor of the primary type.

**Distribution.** Russia Primorskii Territory, East Asia, Southeast Asia.

### 3.2. Keys

**Key to subgenre of *Pedetontus* worldwide**

1. Retractile vesicles restricted to coxites II–V, posterior angles of abdominal sternites ≤90°............................................................... ***Pedetontus* (*Verhoeffilis*) Paclt, 1972**

- Retractile vesicles on coxites II–VI, posterior angles of abdominal sternites obtuse......................................................................***Pedetontus sensu stricto* Silvestri, 1911**

**Key to known species of *Pedetontus* in China**

2. Eye length-to-width ratio > 0.9....................................................................................3

- Eye length-to-width ratio < 0.9..................................................................................10

3. Tarsus with transparent needle-shaped setae..........................................................4

- Tarsus without transparent needle-shaped setae.....................................................8

4. Ratio of distance between inner margins of paired ocelli to combined width of eyes < 0.1.....................................................***P.* (*Verhoeffilis*) *silvestrii* Mendes, 1991**

- Ratio of distance between inner margins of paired ocelli to combined width of eyes > 0.1........................................................................................................................5

5. Ultimate article of maxillary palp (palp VII) longer than penultimate article (palp VI)............................................................................***P.* (*V.*) *formosanus* Silvestri 1943**

- Ultimate article of maxillary palp (palp VII) as long as or shorter than penultimate article (palp VII) ..................................................................................................6

6. Labial palp of male without long hair-like setaea......***P.* (*V.*) *savioi* Silvestri 1936**

- Labial palp of male with long hair-like setaea ..........................................................7

7. Parameres 1+8 articulated..............................***P.* (*V.*) *zhejiangensis* Xue & Yin 1991**

- Parameres 1+7 articulated................***P.* (*V.*) *elegans* Shen, Yang, Ji & Zhang sp. n.**

8. Labial palp of male with long hair-like setae...................................................................................................................................................................................................................***P.* (*V.*) *zhoui* Yu, Zhang & Zhang 2010**

- Labial palp of male without long hair-like setae......................................................9

9. Labial palp of male modified, laterally expanded.....***P.* (*V.*) *issikii* Silvestri 1943**

- Labial palp of male not modified, clavate................***P.* (*V.*) *bianchii* Silvestri 1936**

10. Ratio of distance between inner margins of paired ocelli to combined width of eyes <0.1........................................................................***P.* (*V.*) *uraiensis* Uchida 1965**

- Ratio of distance between inner margins of paired ocelli to combined width of eyes >0.1......................................................................................................................11

11. Tarsus with transparent needle-shaped setae........***P.* (*V.*) *fukiensis* Silvestri 1936**

- Tarsus without transparent needle-shaped setae...................................................12

12. Ovipositor exceeding stylus IX (including supporting spines)............................13

- Ovipositor not exceeding stylus IX (including supporting spines)..................... 15

13. Parameres 1+6 articulated................***P.* (*V.*) *hainanensis* Yu, Zhang & Zhang 2010**

- Parameres more than 1+6 articulated....................................................................... 14

14. Parameres 1+7 articulated......***P.* (*V.*) *nanningensis* Shen, Yang, Ji & Zhang sp. n.**

- Parameres 1+8 articulated.......***P.* (*V.*) *hezhouensis* Shen, Yang, Ji & Zhang sp. n.**

15. Ultimate articles of labial palps (article III) of female clavate, not modified, compound eyes yellowish green............................................................................................................................................................................................***P.* (*V.*) *shenzhenensis* Shen, Yang, Ji & Zhang sp. n.**

- Ultimate articles of labial palps (article III) of female modified, expanded laterally, compound eyes brown...............................................................................................16

16. Ultimate articles of labial palps (article III) of female with more than 30 sensory cones at its apex...................... ***P.* (*V.*) *xanthospilus* Shen, Yang, Ji & Zhang sp. n.**

- Ultimate articles of labial palps (article III) of female without sensory cones at its apex...............................................***P.* (*V.*) *jinxiuensis* Shen, Yang, Ji & Zhang sp. n.**

### 3.3. Description

**Figure 1:**
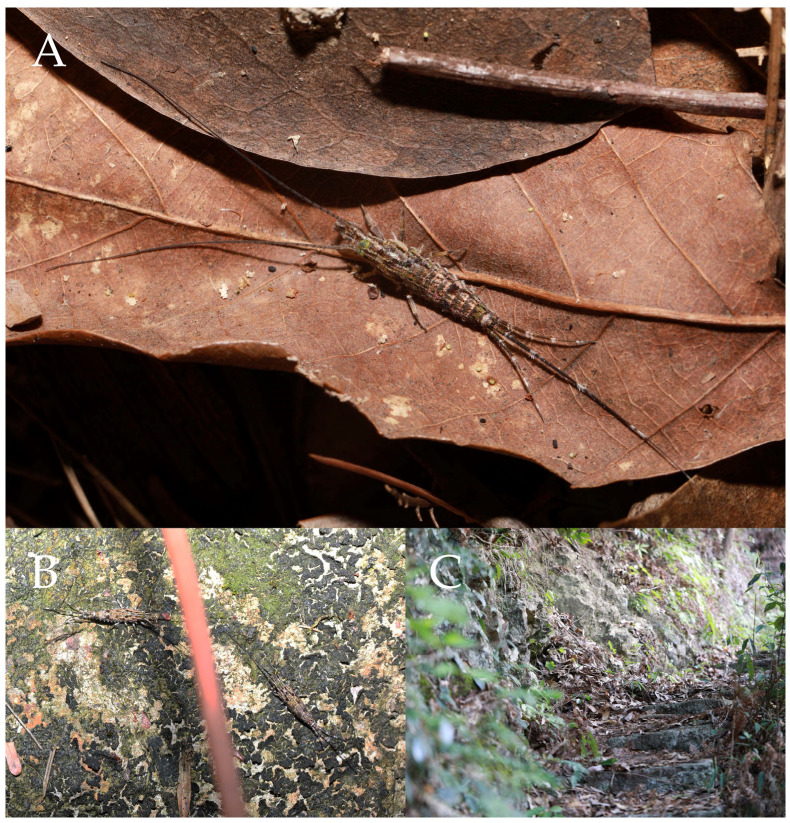
*Pedetontus* (*Verhoeffilis*) *elegans* Shen, Yang, Ji & Zhang sp. n. (**A**) *P.* (*V.*) *elegans* sp. n. in situ. (**B**) Ditto. (**C**) Habitat.

**Figure 2:**
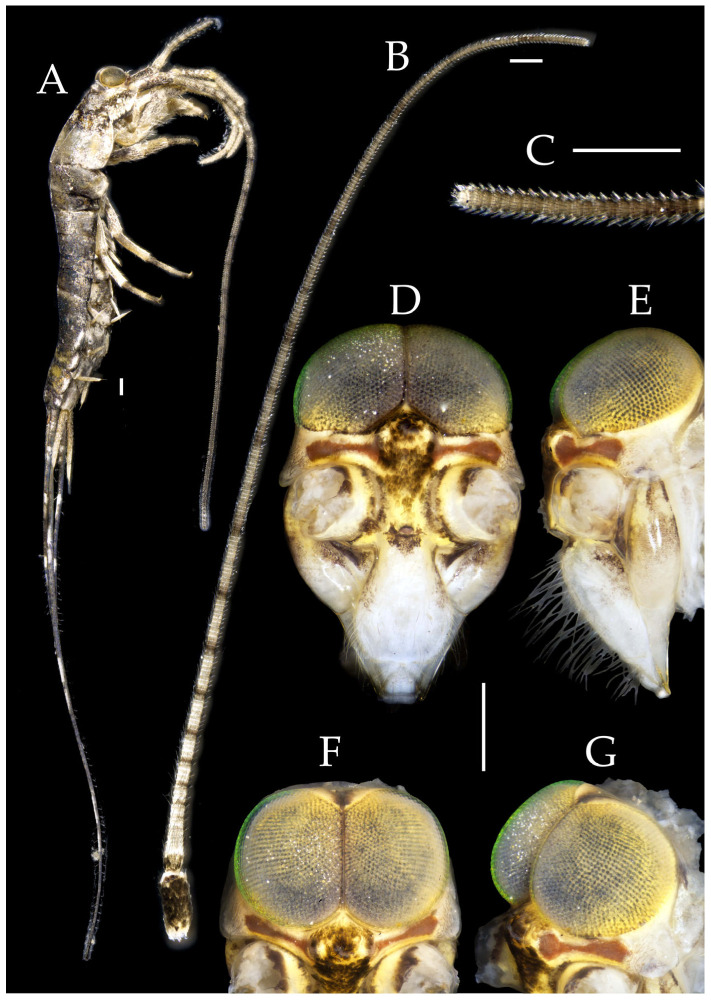
*Pedetontus* (*Verhoeffilis*) *elegans* Shen, Yang, Ji & Zhang sp. n., holotype, male. (**A**) Habitus, lateral view. (**B**) Antenna. (**C**) Terminal chain of flagellum. (**D**) Head, frontal view. (**E**) Ditto, lateral view. (**F**,**G**) Compound eyes and paired ocelli. The white elongated rectangles as the scale bars. Scale bars: 500 μm.

**Figure 3:**
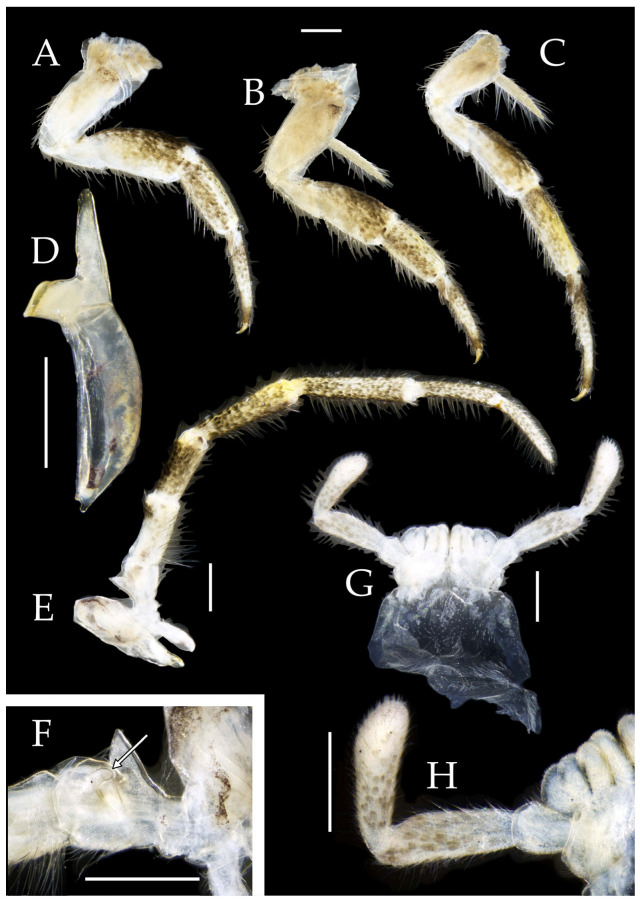
*Pedetontus* (*Verhoeffilis*) *elegans* Shen, Yang, Ji & Zhang sp. n., holotype, male. (**A**–**C**) Foreleg, midleg, and hindleg. (**D**) Mandible. (**E**) Maxilla. (**F**) Maxillary inner protuberance, as indicates by the arrow. (**G**) Labium. (**H**) Labial palp. Scale bars: 500 μm.

**Figure 4:**
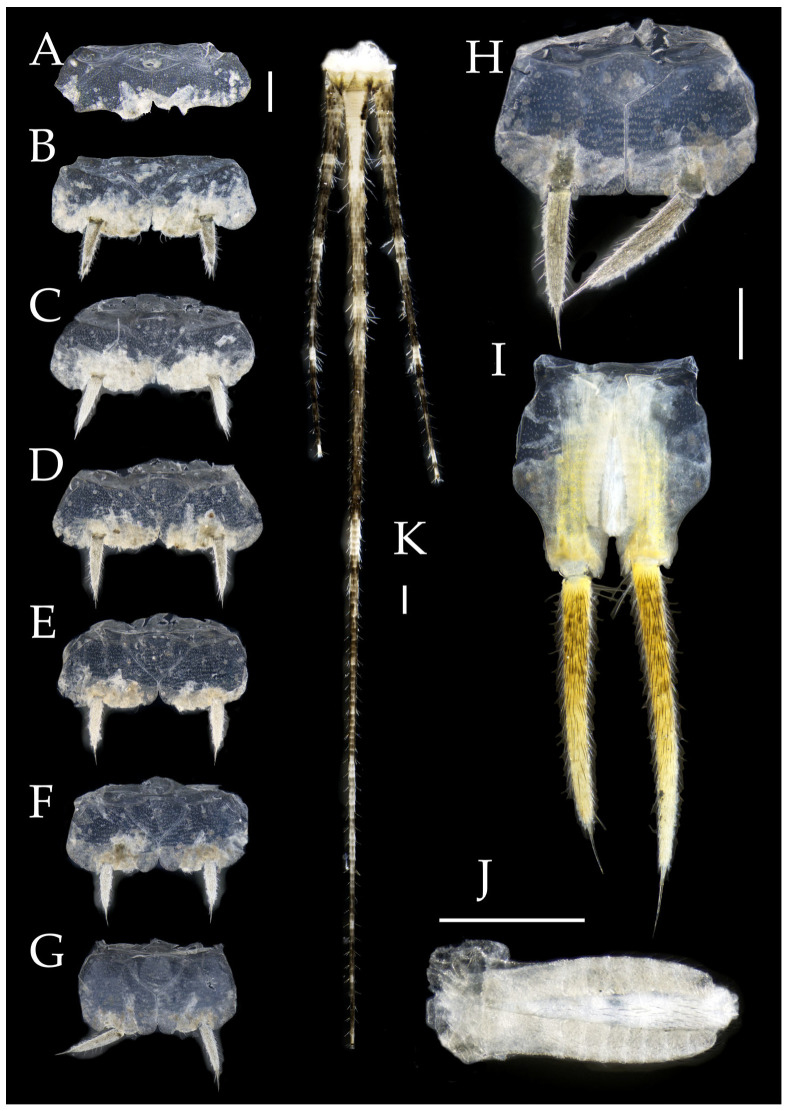
*Pedetontus* (*Verhoeffilis*) *elegans* Shen, Yang, Ji & Zhang sp. n., holotype, male. (**A**–**I**) Abdominal sternites and coxites I–IX. (**J**) Penis and parameres. (**K**) Caudal filament and cerci. Scale bars: 500 μm.

**Figure 5:**
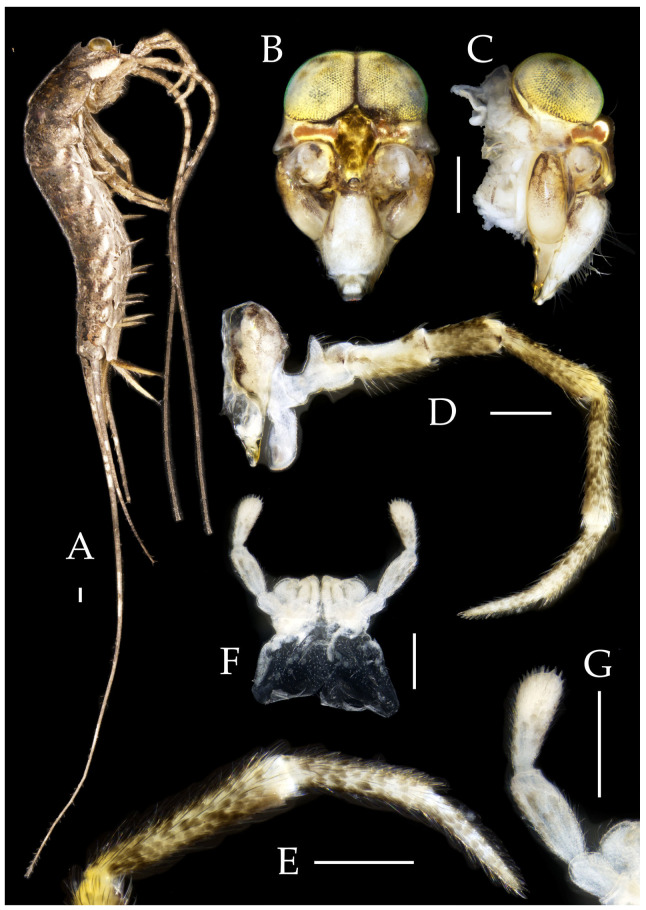
*Pedetontus* (*Verhoeffilis*) *elegans* Shen, Yang, Ji & Zhang sp. n., paratype, female. (**A**) Habitus, lateral view. (**B**) Head, frontal view. (**C**) Ditto, lateral view. (**D**) Maxilla. (**E**) Maxillary palp articles V–VII. (**F**) Labium. (**G**) Labium palp. Scale bars: 500 μm.

**Figure 6:**
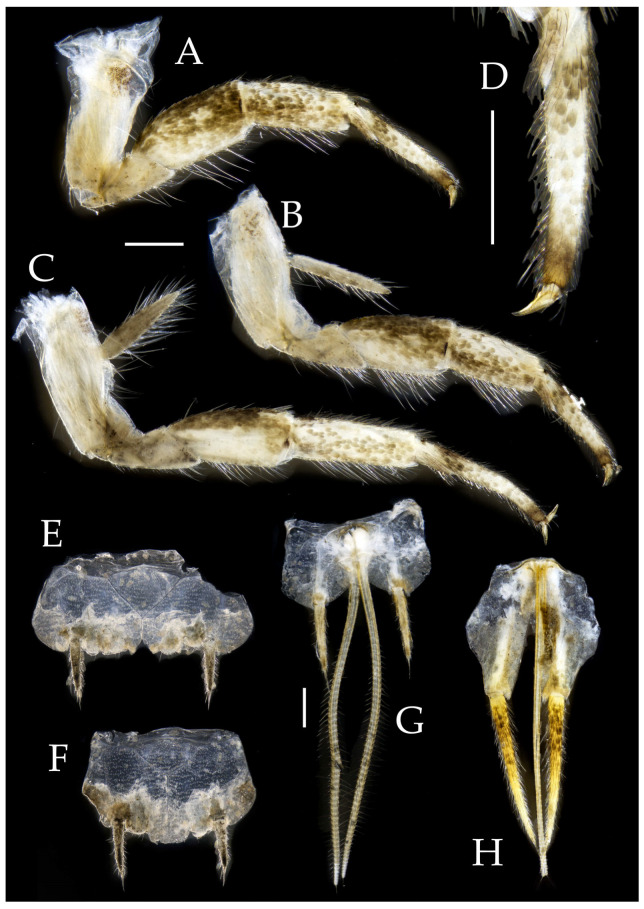
*Pedetontus* (*Verhoeffilis*) *elegans* Shen, Yang, Ji & Zhang sp. n., paratype, female. (**A**–**C**) Foreleg, midleg, and hindleg. (**D**) Foreleg tarsus. (**E–H**) Abdominal sternites and coxites V, VII–IX. Scale bars: 500 μm.

***Pedetontus* (*Verhoeffilis*) *elegans* Shen, Yang, Ji & Zhang sp. n.** (Figure 1, Figure 2, Figure 3, Figure 4, Figure 5 and Figure 6)

Zoobank: urn:lsid:zoobank.org:act:98F98919-E673-4C0A-8FE7-11CCCD2AF8BE

**Type specimens and type locality.** ♂—holotype (in 70% ethanol); 3♂4♀—paratypes (in 70% ethanol). Fengya Valley, Pan’an County, Jinhua City, Zhejiang Province, China (29°04′45″ N 120°37′39″ E, 542 m), in leaf litter and on rocks near signal station (Figure 1), 17.VII.2024, coll. Jia-Yong Zhang, Chen-Yang Shen, Zhi-Qiang Guo, and Jie-Hong Ji. All type specimens deposited in the Animal Herbarium, Zhejiang Normal University, Jinhua, China.

**Description.** Male body length 10.4–11.2 mm, female 11.9 mm. Body covered with scales, light brown, with several white longitudinal stripes, black markings, and a few green markings; caudal filament and cerci with several well-developed white annulate setae. Antennae and median caudal filament approximately 1.6 times body length. Lateral cerci length approximately two-thirds of body length (Figure 1A,B, Figure 2A, and Figure 5A).

**Head.** Compound eyes well-developed, strongly convex, bright yellowish-green (Figure 2D–G and Figure 5B,C). Ratios of eye length to width and contact line length to eye length given in Table 1. Paired ocelli subinferior to compound eyes, reddish-brown, dumbbell-shaped. Ratios of ocellus length to width and distance between inner margins of ocelli to combined width of eyes given in Table 1. Vertex straight, with deep chestnut markings. Frons convex, bearing fine setae, without long bristles. Genae bearing black setae. Clypeus and labrum bearing transparent long setae (Figure 2D,E and Figure 5B,C).

Antennae scaled except on flagellum; flagellum extremely long, with dark annulations and well-developed annulate setae; terminal chains divided into 21–22 segments. Scape length-to-width ratio 2.2–2.33 (Figure 2B).

Mandibles typical, apex with four distal teeth (Figure 3D).

Maxilla basal segment with one processus basalis and one inner protuberance (Figure 3F). Male maxillary palp articles III–VII with well-developed long brush-like setae ventrally; female with long brush-like setae only on article 2. Ultimate article of maxillary palp subequal in length to penultimate article (Figure 3E and Figure 5D,E). Maxillary palp articles V–VII bearing transparent spines dorsally (Figure 3E and Figure 5E); numbers given in Table 2.

Labial palp third article not modified, clavate, apex bearing sensory cone setae; numbers given in Table 2. Male labial palp II and III bearing long hair-like setae; female lacking these (Figure 3G,H and Figure 5F,G).

**Thorax.** Thorax normal. Foreleg femur not expanded, without sensory field; mid- and hindlegs with coxal styli. Coxae, trochanters, femora, and tibiae with long bristles ventrally; tarsi and hind tibiae bearing transparent needle-shaped setae ventrally; numbers given in Table 3. Tibiae bearing black setae dorsally. Pretarsal claw structure normal (Figure 3A–C and Figure 6A–D).

**Abdomen.** Abdominal coxites I, VI, and VII with one pair of retractile vesicles; coxites II–V with two pairs of retractile vesicles (Figure 4A–G and Figure 6E,F). Female coxite VII with inner lobes fused and extending posteriorly (Figure 6F). Angles of posterior angles of abdominal sternites given in Table 4. Ratios of styli length to coxites length on abdominal segment V, styli length to supporting spines length, and basal width to length of sternites given in Table 5. Coxite IX with 2–4+2–4 transparent macrochaetae internally (Figure 4I and Figure 6H). Caudal filament laterally and cerci internally bearing deciduous long setae and supporting spines (Figure 4K).

Coxite VIII without parameres. Coxite IX with penis and parameres (Figure 4I,J). Penis bearing black setae, opening apically, surpassing parameres; parameres 1+7 segmented (Figure 4J). Ovipositor of primary type, surpassing styli (including supporting spines) of coxite IX (Figure 6G,H). Number of segments of anterior gonapophyses and posterior gonapophyses given in Table 6.

**Differential diagnosis.** *Pedetontus* (*Verhoeffilis*) *elegans* sp. n. is most striking due to its extremely large compound eyes and the well-developed ultimate article of the maxillary palp—subequal in length to the penultimate article. *P.* (*V.*) *formosanus* Silvestri, 1943, which also has large eyes and slender maxillary palps; they are morphologically the closest species, but the two can be distinguished by the shape of the lateral ocelli and the length of the article IV of maxillary palp. When viewed laterally from the front, the lateral ocelli of *P.* (*V.*) *elegans* sp. n. are constricted medially, presenting a dumbbell shape, whereas those of *P.* (*V.*) *formosanus* are almost clavate. The maxillary palp IV of *P.* (*V.*) *elegans* sp. n. is subequal in length to palp V, while that of *P.* (*V.*) *formosanus* is distinctly shorter than the palp V. *P.* (*V.*) *diversicornis* Silvestri, 1943, is also similar to *P.* (*V.*) *elegans* sp. n. Key distinguishing characters are antennae and penis length: the former has antennae shorter than the body and a penis subequal in length to the parameres, while the latter has antennae much longer than the body and a penis longer than the parameres.

**Etymology.** The specific epithet “elegans” (Latin adjective meaning “slender” or “graceful”) refers to the species’ slender antennae, elegant markings, and fascinating green eyes. The Chinese name for *P.* (*V.*) *elegans* sp. n. is 秀丽跳蛃.

**Distribution.** Zhejiang Province, China.

**Figure 7:**
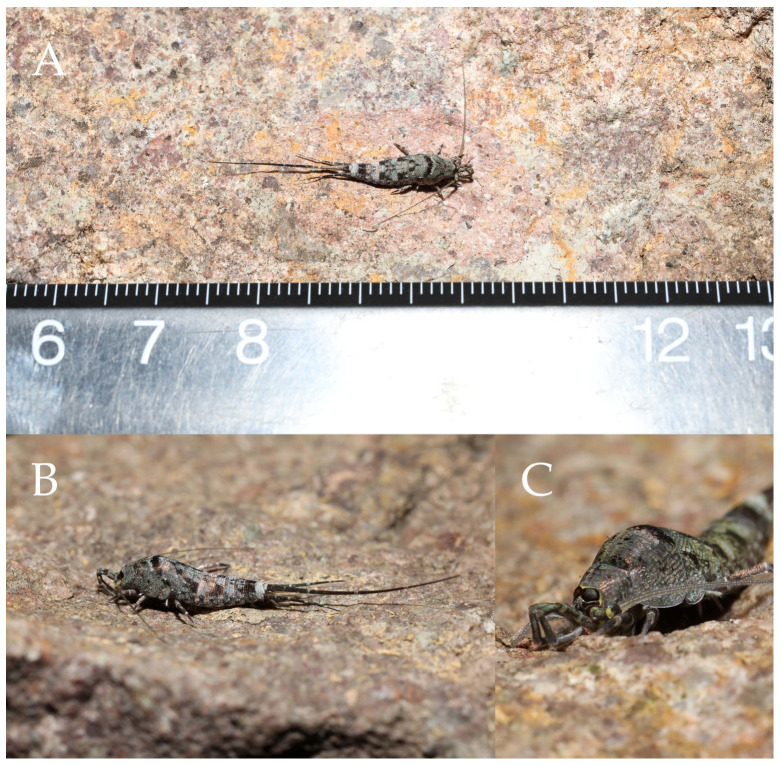
*Pedetontus* (*Verhoeffilis*) *hezhouensis* Shen, Yang, Ji & Zhang sp. n., holotype, female. (**A**–**C**) Dorsal, lateral, and anterolateral view.

**Figure 8:**
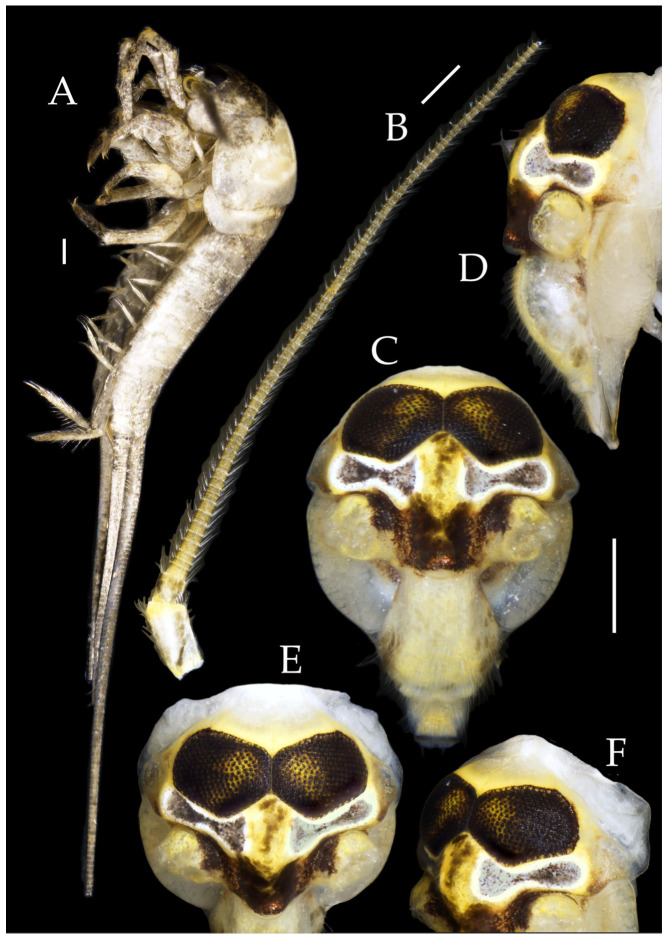
*Pedetontus* (*Verhoeffilis*) *hezhouensis* Shen, Yang, Ji & Zhang sp. n., holotype, female. (**A**) Habitus, lateral view. (**B**) Antenna. (**C**) Head, frontal view. (**D**) Ditto, lateral view. (**E**,**F**) Compound eyes and paired ocelli. Scale bars: 500 μm.

**Figure 9:**
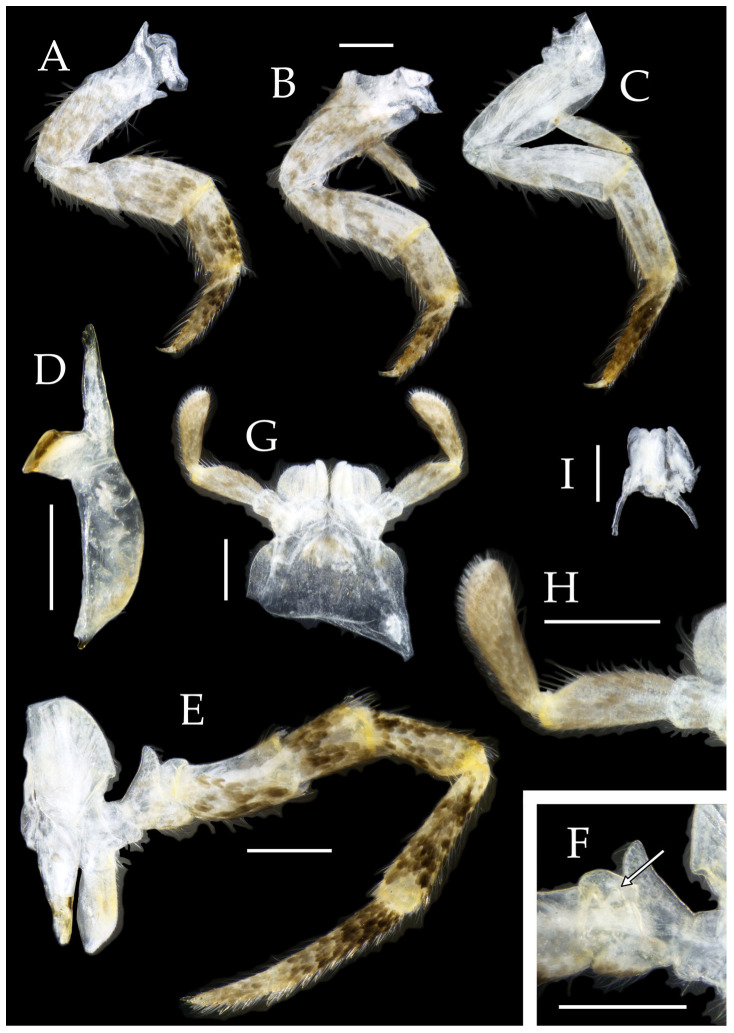
*Pedetontus* (*Verhoeffilis*) *hezhouensis* Shen, Yang, Ji & Zhang sp. n., holotype, female. (**A**–**C**) Foreleg, midleg and hindleg. (**D**) Mandible. (**E**) Maxilla. (**F**) Maxillary inner protuberance, as indicates by the arrow. (**G**) Labium. (**H**) Labial palp. (**I**) Hypopharynx. Scale bars: 500 μm.

**Figure 10:**
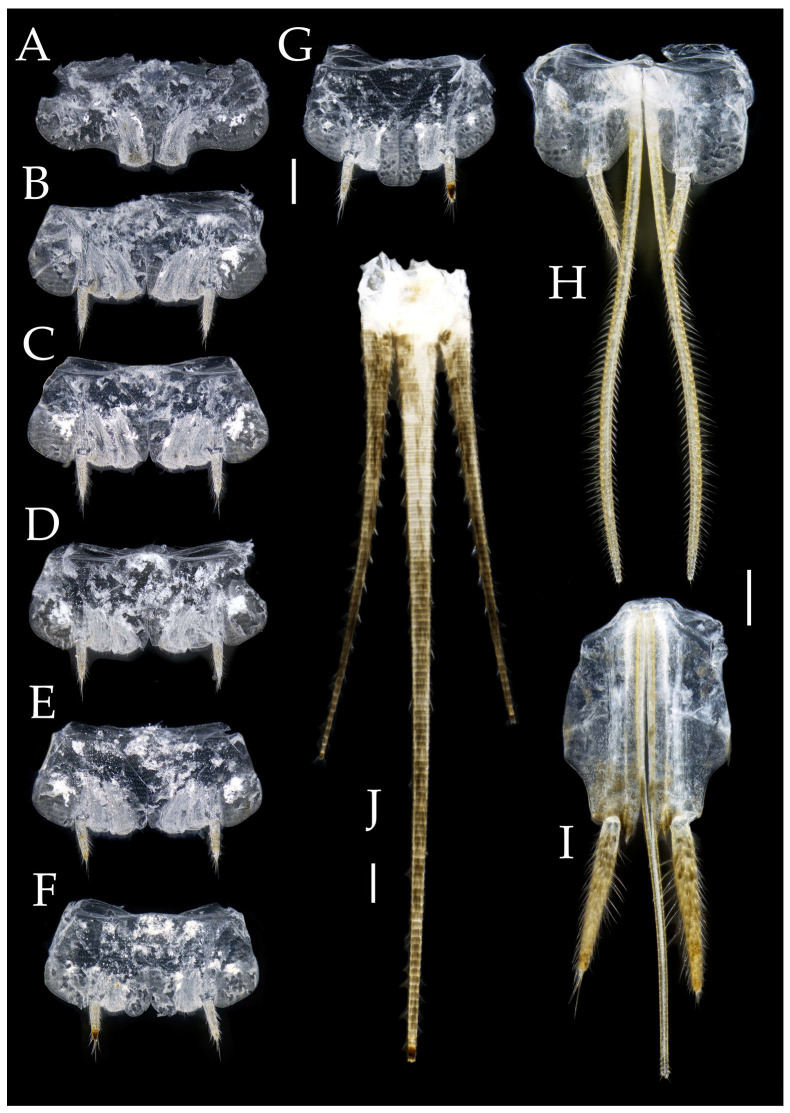
*Pedetontus* (*Verhoeffilis*) *hezhouensis* Shen, Yang, Ji & Zhang sp. n., holotype, female. (**A**–**I**) Abdominal sternites and coxites I–IX. (**J**) Caudal filament and cerci. Scale bars: 500 μm.

**Figure 11:**
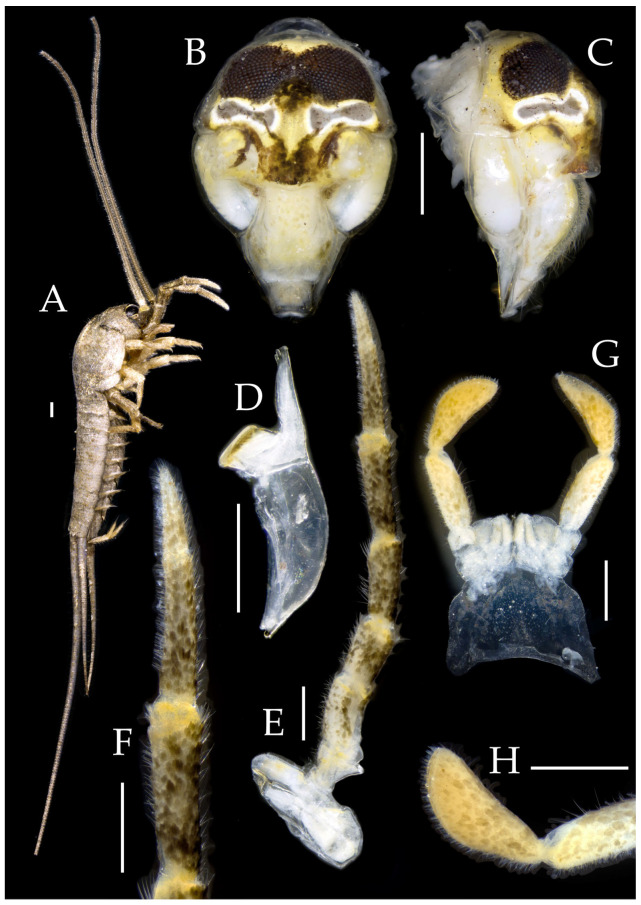
*Pedetontus* (*Verhoeffilis*) *hezhouensis* Shen, Yang, Ji & Zhang sp. n., paratype, male. (**A**) Habitus, lateral view. (**B**) Head, frontal view. (**C**) Ditto, lateral view. (**D**) Mandible. (**E**) Maxilla. (**F**) Maxillary palp articles V–VII. (**G**) Labium. (**H**) Labium palp. Scale bars: 500 μm.

**Figure 12:**
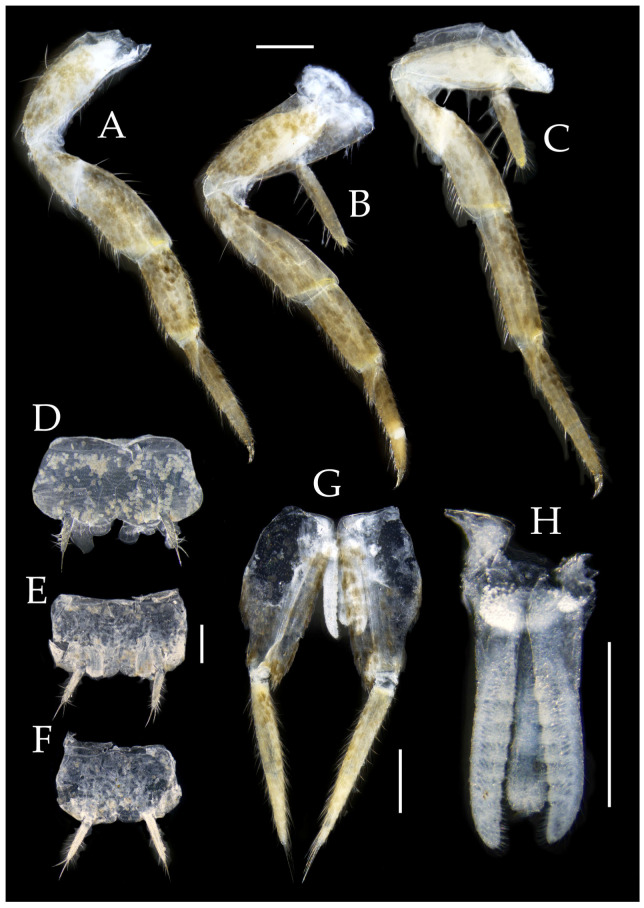
*Pedetontus* (*Verhoeffilis*) *hezhouensis* Shen, Yang, Ji & Zhang sp. n., paratype, male. (**A**–**C**) Foreleg, midleg, and hindleg. (**D**–**G**) Abdominal sternites and coxites V, VII–IX. (**H**) Penis and parameres. Scale bars: 500 μm.

***Pedetontus* (*Verhoeffilis*) *hezhouensis* Shen, Yang, Ji & Zhang sp. n.** (Figure 7, Figure 8, Figure 9, Figure 10, Figure 11 and Figure 12)

Zoobank: urn:lsid:zoobank.org:act:7B7D8D89-1579-40FA-A1EB-04C3B6DEB877

**Type Specimens and Type Locality.** Holotype, 1♀ (in 70% ethanol), paratypes: 1♂6♀ (in 70% ethanol), Huangyao Town, Hezhou City, Guangxi Zhuang Autonomous Region, China (24°14′ N 111°13 E), 18.XII.2024, collected by Jia-Yong Zhang. All type specimens deposited in the Animal Herbarium, Zhejiang Normal University, Jinhua, China.

**Description.** Male body length 7.9 mm, female 7.6–8.9 mm. Body covered with grayish-green scales; posterior part of mesonotum with a dark arched marking; tergites of subsequent segments bearing variously sized paired black spots. Caudal filament and cerci with white annulations. Antennae slightly longer than body length; caudal filament nearly 1.5 times body length; cerci longer than half of body length (Figure 7A,B).

**Head.** Compound eyes small, smooth, dark-colored, with indistinct yellow markings internally (Figure 7C, Figure 8C–F and Figure 11B,C); ratios of eye length to width and contact line length to eye length given in Table 1. Paired ocelli subinferior to compound eyes, black (fading to dark gray when preserved in 70% ethanol), with white borders, shoe-shaped. Ratios of ocellus length to width and distance between inner margins of ocelli to combined width of eyes given in Table 1. Vertex without markings. Frons smooth, with long transparent bristles, covered with scales. Genae without setae. Clypeus and labrum with transparent bristles (Figure 8C–E and Figure 11B,C).

Antennae scaled except on flagellum; flagellum without annulations, bearing well-developed annulate setae; terminal chain divided into 10–13 segments. Scape length-to-width ratio 1.73–1.84 (Figure 8B).

Mandibles typical, apex with four distal teeth (Figure 9D).

Maxillary palp I with one processus basalis and one inner protuberance (Figure 9E,F and Figure 11E). Male maxillary palp articles II–VII with well-developed short brush-like setae ventrally; female without. Ratio of ultimate article to penultimate article length 0.57–0.62 (Figure 9E and Figure 11E,F). Dorsal surface of maxillary palp articles V–VII with transparent spines (Figure 9E and Figure 11E,F); numbers given in Table 2.

Male labial palp yellow, third segment modified, laterally expanded, apex with sensory cones; numbers given in Table 2; ultimate article of female less swollen, clavate. Labial palp without long hair-like setae (Figure 9G,H and Figure 11G,H).

Hypopharynx structure typical (Figure 9I).

**Thorax.** Thorax normal. Foreleg femur not expanded, without sensory field; mid- and hind legs with coxal styli. Ventral surface of legs with long bristles, without needle-shaped setae. Pretarsal claw structure normal (Figure 9A–C and Figure 12A–C).

**Abdomen.** Abdominal coxites I, VI, and VII with one pair of retractile vesicles; coxites II–V with two pairs of retractile vesicles (Figure 10A–G and Figure 12D,E); inner lobes of female coxite VII fused and posteriorly extended (Figure 10G). Ratios of styli length to coxites length on abdominal segment V, styli length to supporting spines length, and basal width to length of sternites given in Table 5. Coxite IX medially with 4–8+4–8 transparent macrochaetae (Figure 10I and Figure 12G). Caudal filament laterally and cerci medially with deciduous long hairs and supporting spines (Figure 10J).

Coxite VIII without parameres, coxite IX with penis and parameres (Figure 12F,G). Penis opening apically, slightly shorter than parameres; parameres 1+8 segmented (Figure 10H). Ovipositor of primary type, surpassing styli (including supporting spines) of coxite IX (Figure 10H,I). Number of segments of anterior gonapophyses and posterior gonapophyses given in Table 6.

**Differential diagnosis.** *Pedetontus* (*Verhoeffilis*) *hezhouensis* sp. n. is morphologically most similar to *P.* (*V.*) *sauterii* Silvestri, 1943, from Taiwan, China. They differ primarily in the length of the contact line of the eyes: in the former, the ratio of this structure’s length to the length of the eye is <0.4, while in the latter, it is >0.4.

**Etymology.** The species is named after its type locality, Hezhou City. The Chinese name for *P.* (*Verhoeffilis*) *hezhouensis* sp. n. is 贺州跳蛃.

**Distribution.** Hezhou City, Guangxi Zhuang Autonomous Region, China.

**Figure 13:**
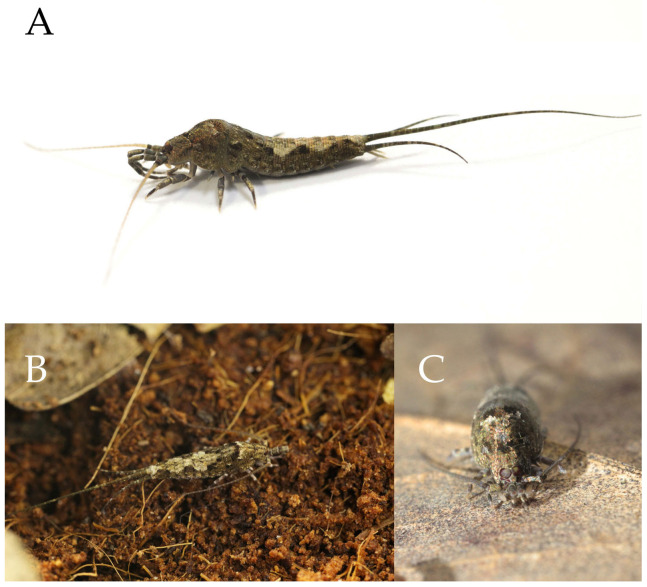
*Pedetontus* (*Verhoeffilis*) *jinxiuensis* Shen, Yang, Ji & Zhang sp. n. (**A–C**) Lateral, dorsal, and anterior view.

**Figure 14:**
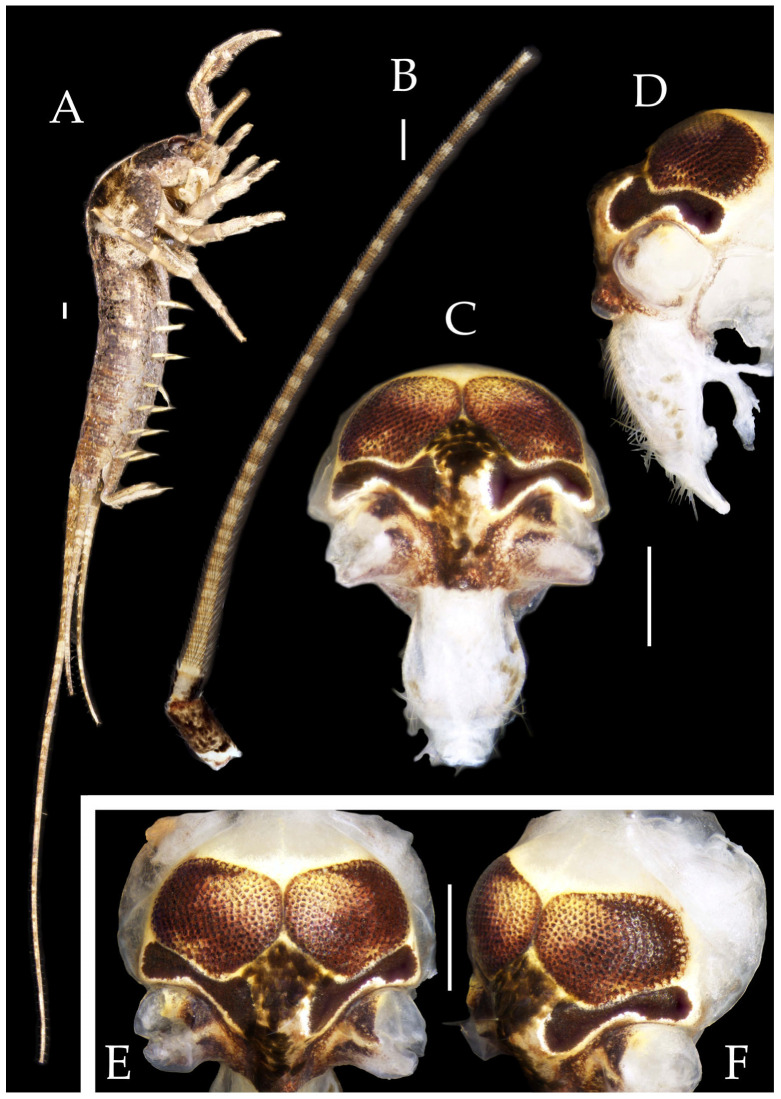
*Pedetontus* (*Verhoeffilis*) *jinxiuensis* Shen, Yang, Ji & Zhang sp. n., holotype, male. (**A**) Habitus, lateral view. (**B**) Antenna. (**C**) Head, frontal view. (**D**) Ditto, lateral view. (**E**,**F**) Compound eyes and paired ocelli. Scale bars: 500 μm.

**Figure 15:**
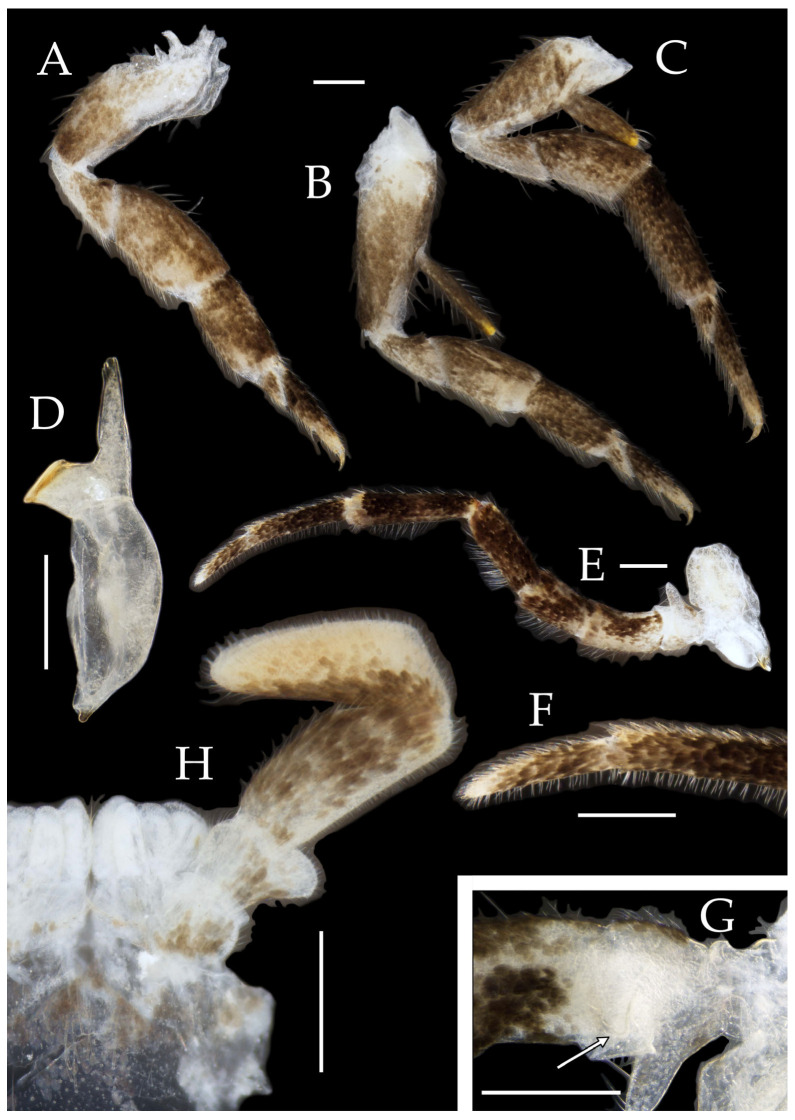
*Pedetontus* (*Verhoeffilis*) *jinxiuensis* Shen, Yang, Ji & Zhang sp. n., holotype, male. (**A**–**C**) Foreleg, midleg, and hindleg. (**D**) Mandible. (**E**) Maxilla. (**F**) Maxillary palp article VI and VII. (**G**) Maxillary inner protuberance, as indicates by the arrow. (**H**) Labium. Scale bars: 500 μm.

**Figure 16:**
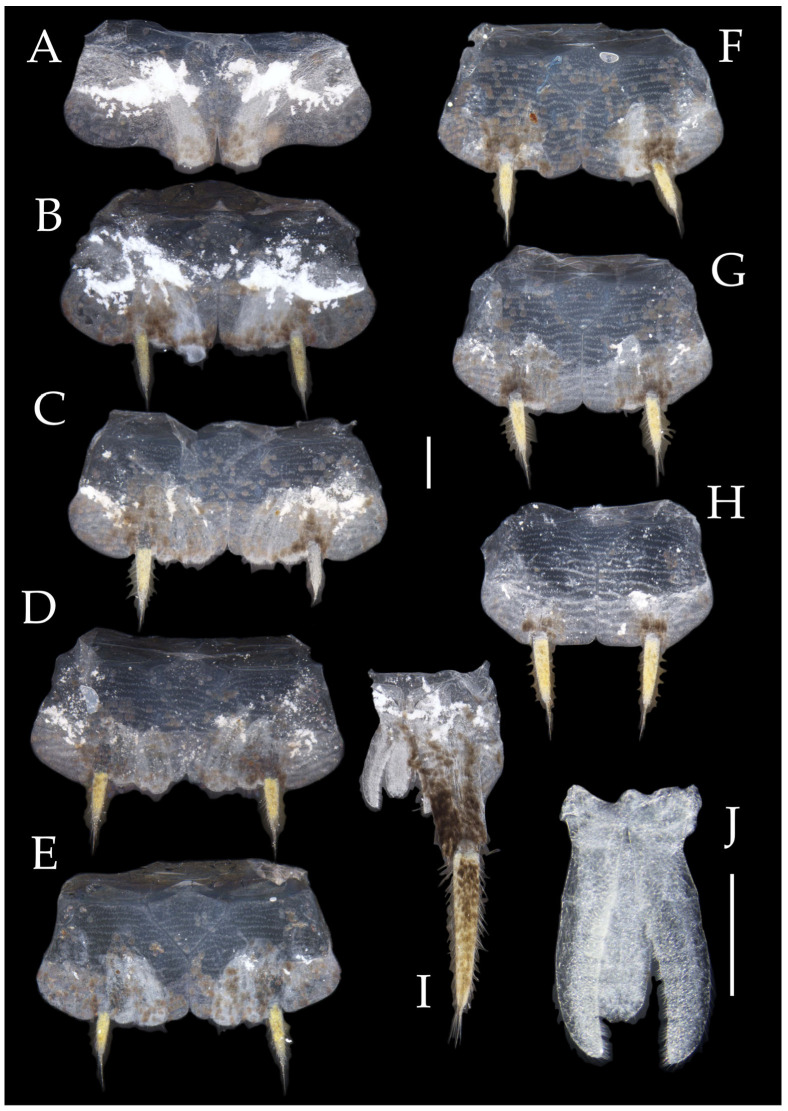
*Pedetontus* (*Verhoeffilis*) *jinxiuensis* Shen, Yang, Ji & Zhang sp. n., holotype, male. (**A**–**I**) Abdominal sternites and coxites I–IX. (**J**) Penis and parameres. Scale bars: 500 μm.

**Figure 17:**
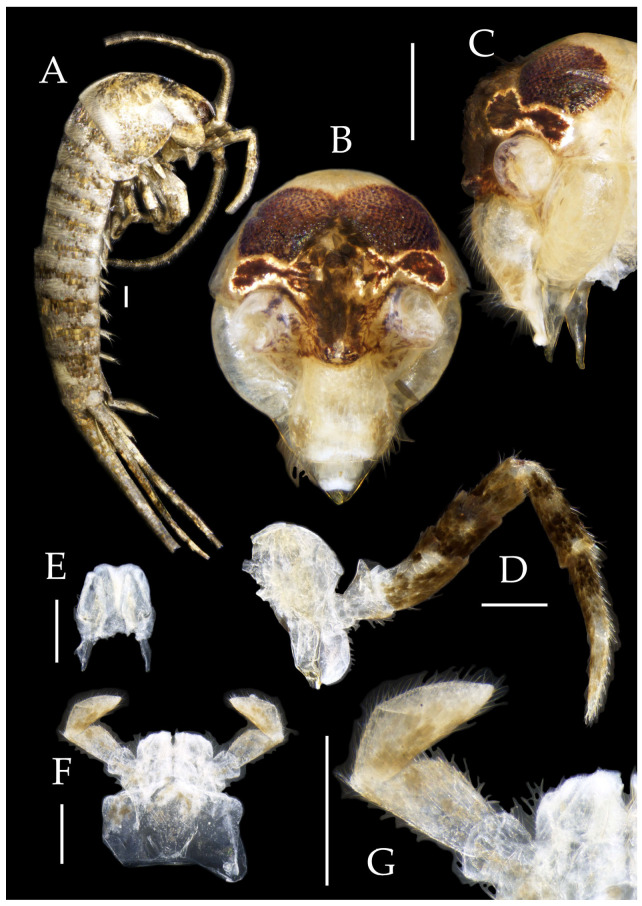
*Pedetontus* (*Verhoeffilis*) *jinxiuensis* Shen, Yang, Ji & Zhang sp. n., paratype, female. (**A**) Habitus, lateral view. (**B**) Head, frontal view. (**C**) Ditto, lateral view. (**D**) Maxilla. (**E**) Hypophraynx. (**F**) Labium. (**G**) Labium palp. Scale bars: 500 μm.

**Figure 18:**
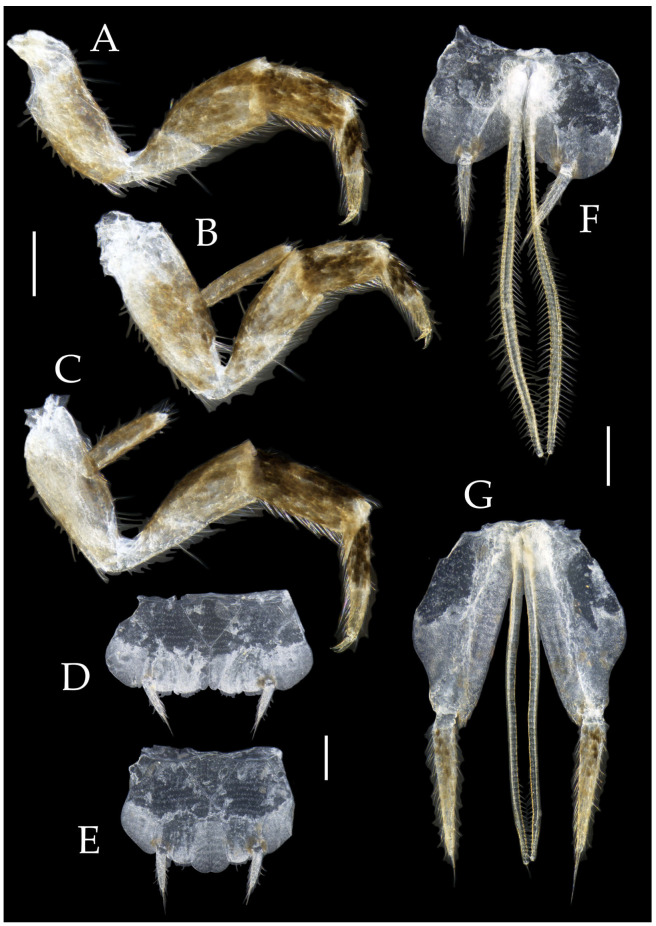
*Pedetontus* (*Verhoeffilis*) *jinxiuensis* Shen, Yang, Ji & Zhang sp. n., paratype, female. (**A**–**C**) Foreleg, midleg, and hindleg. (**D**–**G**) Abdominal sternites and coxites V, VII–IX. Scale bars: 500 μm.

***Pedetontus* (*Verhoeffilis*) *jinxiuensis* Shen, Yang, Ji & Zhang sp. n.** (Figure 13, Figure 14, Figure 15, Figure 16, Figure 17 and Figure 18)

Zoobank: urn:lsid:zoobank.org:act:4C21B2F2-5BD0-4D46-BD78-7A78A941DCA0

**Type Specimens and Type Locality.** Holotype, 1♂ (in 70% ethanol), paratypes: 15♂2♀ (in 70% ethanol), China, Guangxi Zhuang Autonomous Region, Jinxiu County, Dayao Mountain (24°6′4.88″ N, 110°9′37.69″ E, 947 m), 1.V.2023, collected by Wen-Yong Feng. All type specimens deposited in the Animal Herbarium, Zhejiang Normal University, Jinhua, China.

**Description.** Male body length 11.2 mm, female 9.7 mm. Body covered with dark brown scales; mesonotum with an arched dark marking; subsequent nota bearing paired dark patches; abdominal nota patterned with three longitudinal pale diamond-shaped markings. Antennae equal to or slightly longer than body length; caudal filament nearly 1.5 times body length; cerci about half as long as body length (Figure 13A,B).

**Head.** Compound eyes smooth, deep reddish-brown, with indistinct yellow blotches (Figure 14C–F and Figure 17B,C); ratios of eye length to width and contact line length to eye length given in Table 1. Ocelli dark brown with white borders, dumbbell-shaped. Ratios of ocellus length to width and distance between inner margins of ocelli to combined width of eyes given in Table 1. Vertex without markings. Frons convex, with long transparent bristles, scaled. Genae without setae. Clypeus and labrum with transparent bristles (Figure 14C–F and Figure 17B,C).

Antennae scaled except on flagellum; flagellum with pale annulations, bearing well-developed annulate setae; terminal chain divided into 8–10 segments. Scape length-to-width ratio 1.86–1.91 (Figure 14B).

Mandibles typical, apex with four distal teeth (Figure 15D).

Maxillary palp I with one processus basalis and one inner protuberance (Figure 15E,G and Figure 17D). Male maxillary palp articles II–VII with well-developed short brush-like setae ventrally; female without. Ratio of ultimate article to penultimate article length 0.69–0.85. Dorsal surface of maxillary palp articles V–VII with transparent spines (Figure 15E,F and Figure 17D); numbers given in Table 2.

Labial palp third segment modified, extremely laterally expanded, apex with sensory cones; numbers given in Table 2; ultimate article of female less swollen, apex without sensory cones. Labial palp without long hair-like setae (Figure 15H and Figure 17F,G).

Hypopharynx structure typical (Figure 17E).

**Thorax.** Thorax normal. Foreleg femur not expanded, without sensory field; mid- and hind legs with coxal styli, apex of coxal styli yellow. Ventral surface of legs with long bristles, without needle-shaped setae. Pretarsal claw structure normal (Figure 15A–C and Figure 18A–C).

**Abdomen.** Abdominal coxites I, VI, and VII with one pair of retractile vesicles; coxites II–V with two pairs of retractile vesicles; coxites I–IX each with a pair of yellow styli (Figure 16A–I and Figure 18D–G); inner lobes of female coxite VII fused and posteriorly extended (Figure 18E). Ratios of styli length to coxites length on abdominal segment V, styli length to supporting spines length, and basal width to length of sternites given in Table 5. Coxite IX medially with 3–4+3–4 transparent macrochaetae (Figure 16I and Figure 18G). Caudal filament laterally and cerci medially with deciduous long hairs and supporting spines (Figure 13B and Figure 14A).

Coxite VIII without parameres. Coxite IX with penis and parameres (Figure 16I,J). Penis opening apically, slightly shorter than parameres; parameres 1+7 segmented (Figure 16J). Ovipositor of primary type, surpassing styli (including supporting spines) of coxite IX (Figure 18G). Number of segments of anterior gonapophyses and posterior gonapophyses given in Table 6.

**Differential diagnosis.** *Pedetontus* (*Verhoeffilis*) *jinxiuensis* sp. n. is morphologically very similar to *P.* (*V.*) *xanthospilus* sp. n. from Zhaoqing, Guangdong Province, but can be distinguished by the form of the labial palps: the former has a highly modified ultimate article (width significantly greater than length), with the anterolateral part of article I in males also laterally expanded. Additionally, the apex of the female ultimate article lacks sensory cones—an extremely rare feature. In contrast, although the labial palps of *P.* (*V.*) *xanthospilus* sp. n. are also swollen, they are far less modified than in *P.* (*V.*) *jinxiuensis* sp. n.; article I lacks specialization, and both sexes possess sensory cones at the apex of the ultimate article.

**Etymology.** The species is named after its type locality, Jinxiu County. The Chinese name for *P.* (*V.*) *jinxiuensis* is 金秀跳蛃.

**Distribution.** Jinxiu County, Guangxi Zhuang Autonomous Region, China.

**Figure 19:**
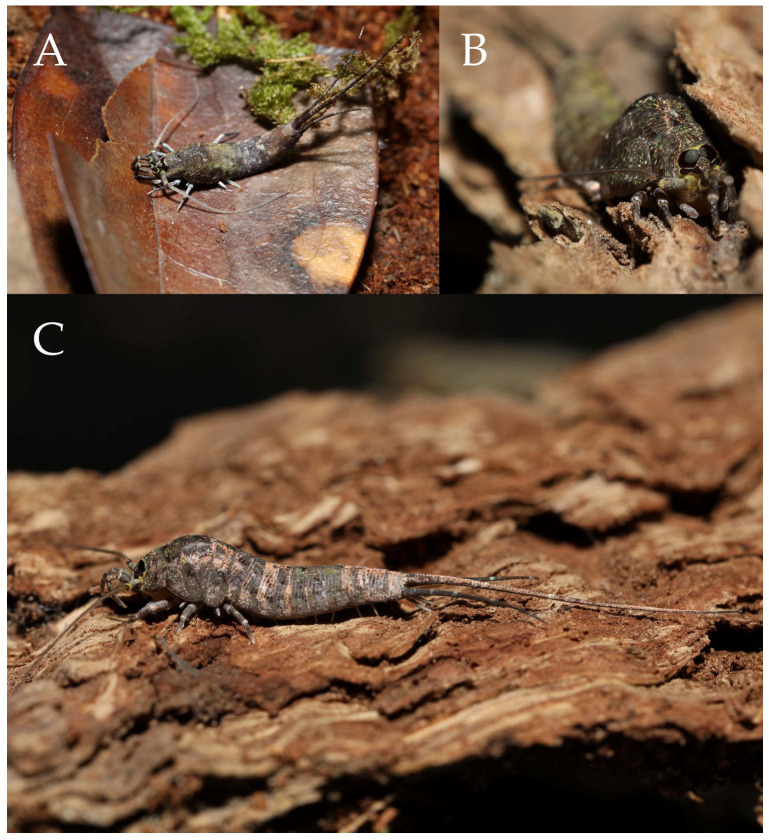
*Pedetontus* (*Verhoeffilis*) *nanningensis* Shen, Yang, Ji & Zhang sp. n. (**A**–**C**) Dorsal, anterolateral and lateral view.

**Figure 20:**
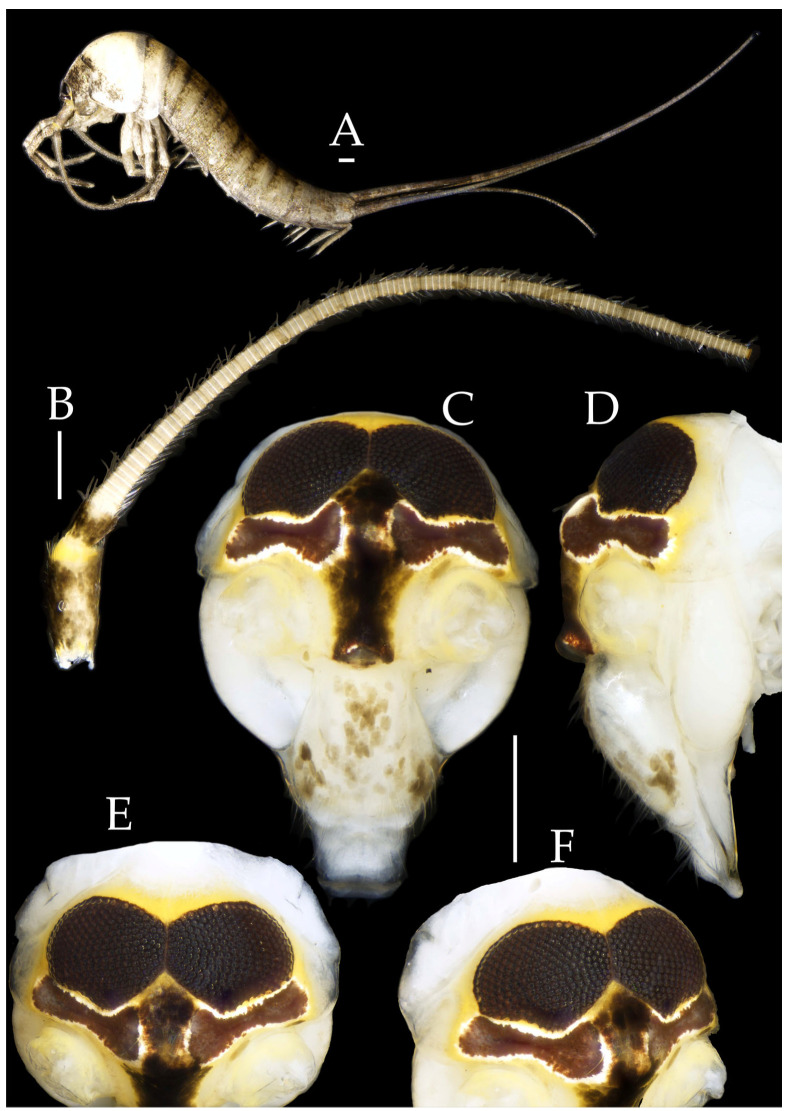
*Pedetontus* (*Verhoeffilis*) *nanningensis* Shen, Yang, Ji & Zhang sp. n., holotype, male. (**A**) Habitus, lateral view. (**B**) Antenna. (**C**) Head, frontal view. (**D**) Ditto, lateral view. (**E**,**F**) Compound eyes and paired ocelli. Scale bars: 500 μm.

**Figure 21:**
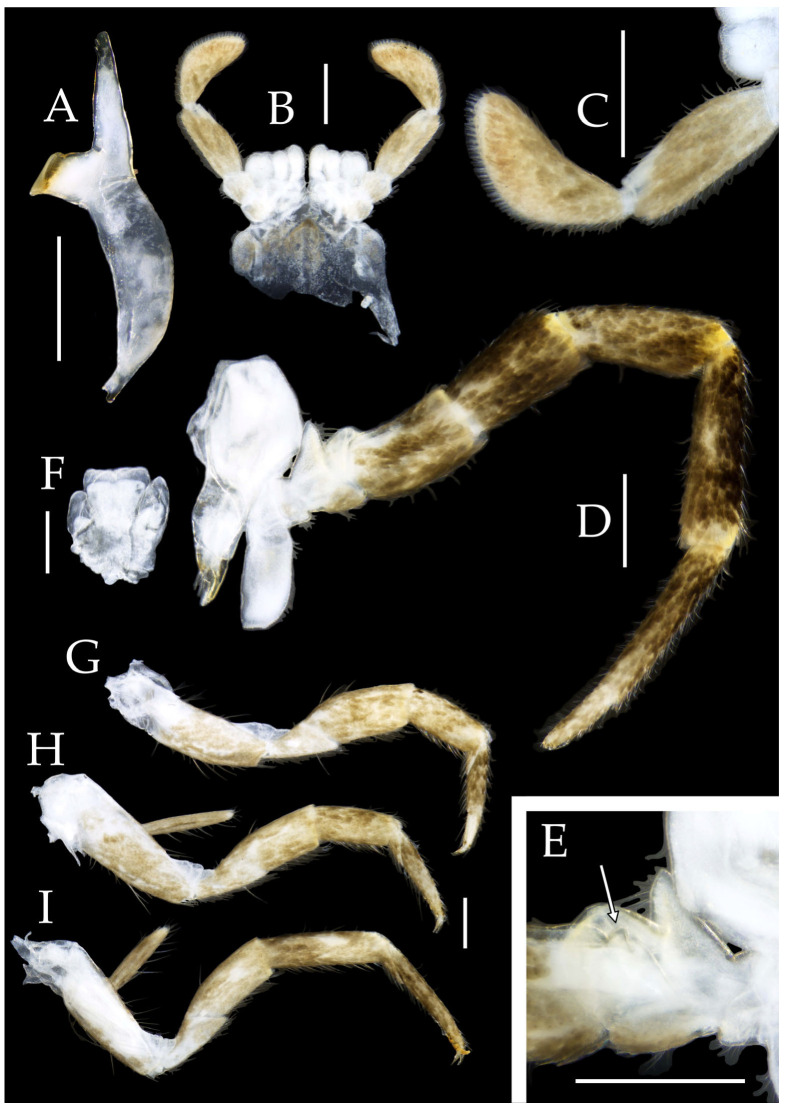
*Pedetontus* (*Verhoeffilis*) *nanningensis* Shen, Yang, Ji & Zhang sp. n., holotype, male. (**A**) Mandible (**B**) Labium. (**C**) Labial palp (**D**) Maxilla. (**E**) Maxillary inner protuberance, as indicates by the arrow. (**F**) Hypopharynx. (**G**–**I**) Foreleg, midleg, and hindleg. Scale bars: 500 μm.

**Figure 22:**
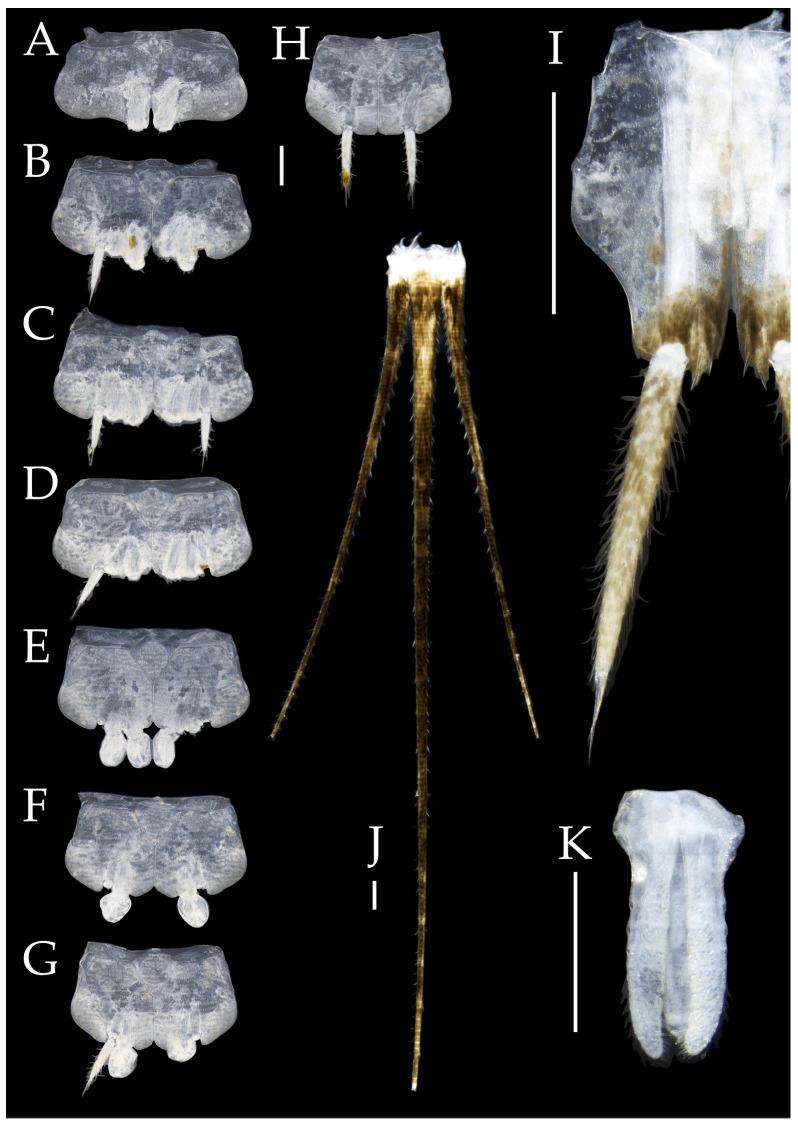
*Pedetontus* (*Verhoeffilis*) *nanningensis* Shen, Yang, Ji & Zhang sp. n., holotype, male. (**A**–**I**) Abdominal sternites and coxites I–IX. (**J**) Caudal filament and cerci. (**K**) Penis and parameres. Scale bars: 500 μm.

**Figure 23:**
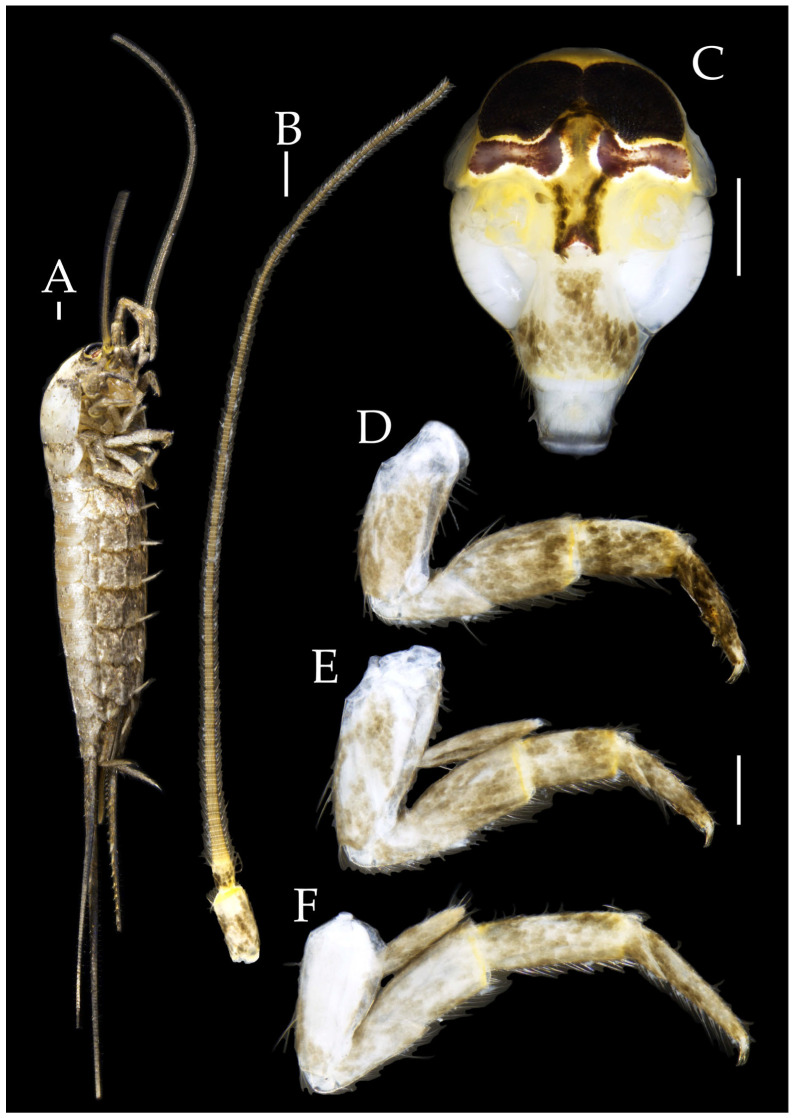
*Pedetontus* (*Verhoeffilis*) *nanningensis* Shen, Yang, Ji & Zhang sp. n., paratype, female. (**A**) Habitus, lateral view. (**B**) Antenna. (**C**) Head, frontal view. (**D**–**F**) Foreleg, midleg, and hindleg. Scale bars: 500 μm.

**Figure 24:**
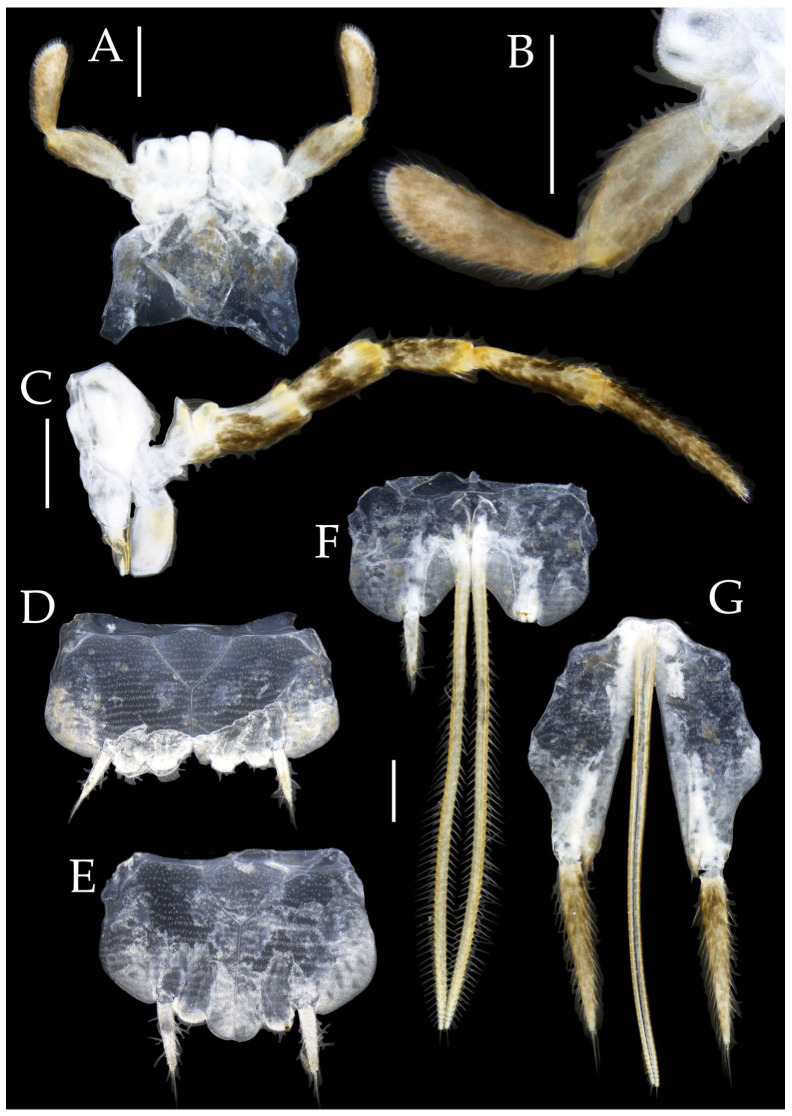
*Pedetontus* (*Verhoeffilis*) *nanningensis* Shen, Yang, Ji & Zhang sp. n., paratype, female. (**A**) Labium. (**B**) Labial palp. (**C**) Maxilla. (**D**–**G**) Abdominal sternites and coxites V, VII–IX. Scale bars: 500 μm.

***Pedetontus* (*Verhoeffilis*) *nanningensis* Shen, Yang, Ji & Zhang sp. n.** (Figure 19, Figure 20, Figure 21, Figure 22, Figure 23 and Figure 24)

Zoobank: urn:lsid:zoobank.org:act:4D83A441-4098-4384-B529-75E5B3D303A4

**Type Specimens and Type Locality.** Holotype, 1♂ (in 70% ethanol), paratypes: 4♂ 6♀ (in 70% ethanol), collected from Jiangnan District, Nanning City, Guangxi Zhuang Autonomous Region, China, 18 II.2025, by Jia-Yong Zhang. All type specimens deposited in the Animal Herbarium, Zhejiang Normal University, Jinhua, China.

**Description.** Male body length 10.6 mm, female 11.6 mm. Body covered with grayish-green scales. Antennae slightly shorter than body length; caudal filament slightly longer than body length; cerci about three-quarters as long as body length (Figure 19A,C).

**Head.** Compound eyes smooth, black, without markings (Figure 19B, Figure 20C–F and Figure 23C); ratios of eye length to width and contact line length to eye length given in Table 1. Ocelli dark brown with white borders, dumbbell-shaped. Ratios of ocellus length to width and distance between inner margins of ocelli to combined width of eyes given in Table 1. Vertex without markings. Frons convex, with long transparent bristles, scaled. Genae without bristles. Clypeus and labrum with transparent bristles (Figure 20C–F and Figure 23C).

Antennae scaled except on flagellum; flagellum almost without dark annulations, bearing well-developed annulate setae; terminal chain divided into 14 segments. Scape length-to-width ratio 1.97–2.11 (Figure 20B and Figure 23B).

Mandibles typical, apex with four distal teeth (Figure 21A).

Maxillary palp I with one processus basalis and one inner protuberance (Figure 21D,E and Figure 24C). Maxillary palp articles without short brush-like setae ventrally. Ratio of ultimate article to penultimate article length 0.45–0.52. Dorsal surface of maxillary palp articles V–VII with transparent spines (Figure 21D and Figure 24C); numbers given in Table 2.

Hypopharynx structure typical (Figure 21F).

Male labial palp third segment modified, laterally expanded, apex with sensory cones; numbers given in Table 2; ultimate article of female almost unswollen, clavate. Labial palp without long hair-like setae (Figure 21B,C and Figure 24A,B).

**Thorax.** Thorax normal. Foreleg without sensory field or coxal styli; mid- and hind legs with coxal styli. Ventral surface of legs with long bristles, without needle-shaped setae. Pretarsal claw structure normal (Figure 21G–I and Figure 23D–F).

**Abdomen.** Abdominal coxites I, VI, and VII with one pair of retractile vesicles; coxites II–V with two pairs of retractile vesicles (Figure 22A–G and Figure 24D,E); inner lobes of female coxite VII fused and posteriorly extended (Figure 24E). Ratios of styli length to coxites length on abdominal segment V, styli length to supporting spines length, and basal width to length of sternites given in Table 5. Coxite IX medially with 6+6 transparent macrochaetae (Figure 22I and Figure 24G). Caudal filament laterally and cerci medially with deciduous long hairs and supporting spines (Figure 22J).

Coxite VIII without parameres. Coxite IX with penis and parameres (Figure 22H,I). Penis opening apically, slightly shorter than parameres; parameres 1+7 segmented (Figure 22K). Ovipositor of primary type, narrowly exceeding styli (including supporting spines) of coxite IX (Figure 24F,G). Number of segments of anterior gonapophyses and posterior gonapophyses given in Table 6.

**Differential diagnosis.** *Pedetontus* (*Verhoeffilis*) *nanningensis* sp. n. is morphologically most similar to *P.* (*V.*) *gershneri* Allen, 1995, from Arkansas, USA. They can be distinguished by the maxillary palps: the former lacks brush-like setae ventrally on the male maxillary palps, whereas the latter possesses brush-like setae ventrally on articles V–VII. Additionally, the ultimate article of the maxillary palp in *P.* (*V.*) *nanningensis* sp. n. is only half the length of the penultimate article, while in *P.* (*V.*) *gershneri* it exceeds three-quarters the length of the penultimate article.

**Etymology.** The species is named after its type locality, Nanning City. The Chinese name for *P.* (*V.*) *nanningensis* sp. n. is 南宁跳蛃.

**Distribution.** Guangxi Zhuang Autonomous Region, China.

**Figure 25:**
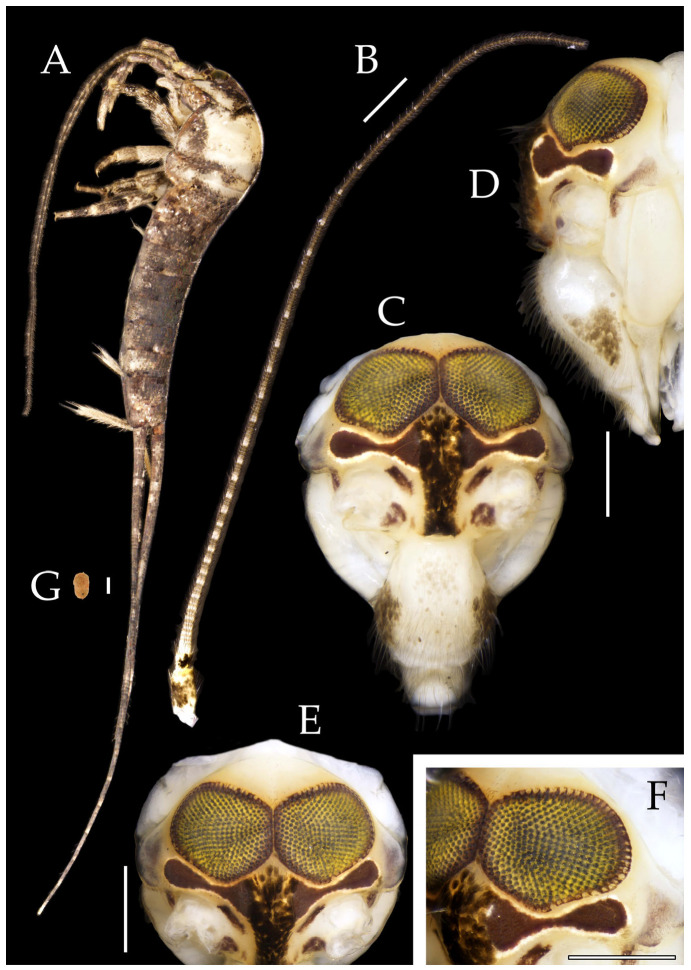
*Pedetontus* (*Verhoeffilis*) *shenzhenensis* Shen, Yang, Ji & Zhang sp. n., holotype, female. (**A**) Habitus, lateral view. (**B**) Antenna. (**C**) Head, frontal view. (**D**) Ditto, lateral view. (**E**,**F**) Compound eyes and paired ocelli. (**G**) Excrement. Scale bars: 500 μm.

**Figure 26:**
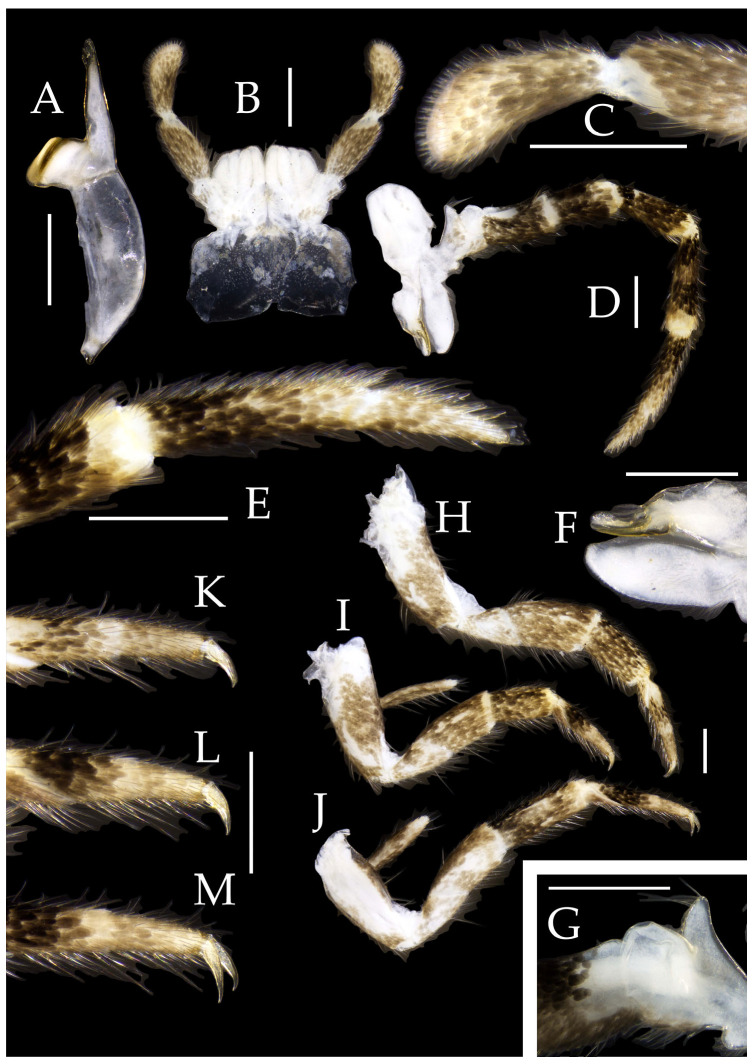
*Pedetontus* (*Verhoeffilis*) *shenzhenensis* Shen, Yang, Ji & Zhang sp. n., holotype, female. (**A**) Mandible. (**B**) Labium. (**C**) Labial palp. (**D**) Maxilla. (**E**) Maxillary palp article VI and VII. (**F**) Lacinia and galea. (**G**) Maxillary inner protuberance. (**G**) Labium. (**H**–**J**) Foreleg, midleg, and hindleg. (**K**–**M**) Foreclaw, midclaw, and hindclaw. Scale bars: 500 μm.

**Figure 27:**
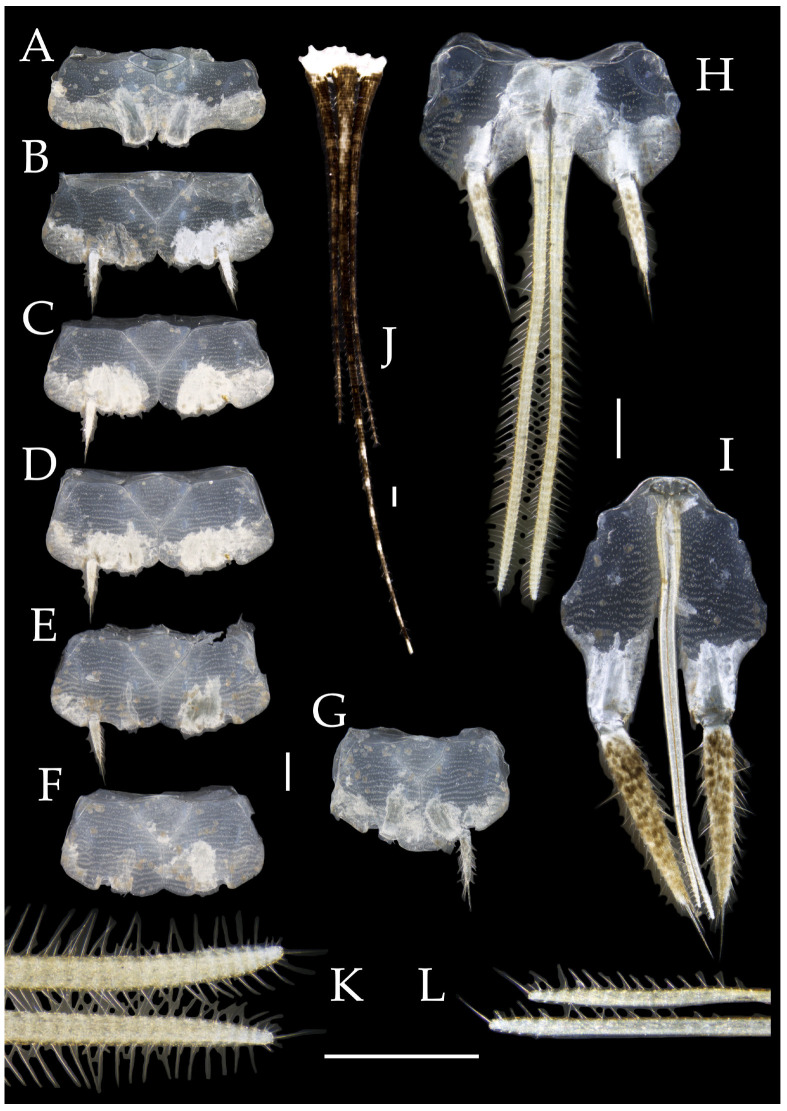
*Pedetontus* (*Verhoeffilis*) *shenzhenensis* Shen, Yang, Ji & Zhang sp. n., holotype, female. (**A**–**I**) Abdominal sternites and coxites I–IX. (**J**) Caudal filament and cerci. (**K**) Apex of anterior gonapophyses. (**L**) Apex of posterior gonapophyses. Scale bars: 500 μm.

***Pedetontus shenzhenensis* Shen, Yang, Ji & Zhang sp. n.** (Figure 25, Figure 26 and Figure 27)

Zoobank: urn:lsid:zoobank.org:act:D809BC47-4BED-4B63-993D-31267071EE31

**Type Specimens and Type Locality.** Holotype, 1♀ (in 70% ethanol), paratype, 1♀ collected from Yangtai Mountain, Shenzhen City, Guangdong Province, China (22°39′57″ N 113°58′29″ E, 129 m), 3.IV.2023, by Yong-Ying Ruan All type specimens deposited in the Animal Herbarium, Zhejiang Normal University, Jinhua, China.

**Description.** Female 11.2 mm. Body covered with brown scales (in 70% ethanol). Antennae longer than body length; caudal filament approximately 1.5 times body length; cerci slightly shorter than body length (Figure 25A).

**Head.** Compound eyes smooth, yellowish-green with maroon borders (Figure 25C–F); ratios of eye length to width and contact line length to eye length given in Table 1. Ocelli dark brown with white borders, dumbbell-shaped. Ratios of ocellus length to width and distance between inner margins of ocelli to combined width of eyes given in Table 1. Vertex without markings, bearing minute black setae arranged longitudinally. Frons convex, with long transparent bristles and minute black setae, scaled. Genae with black setae near compound eyes. Clypeus and labrum with transparent setae (Figure 25C–F).

Antennae scaled except on flagellum; flagellum with distinct pale annulations, bearing well-developed annulate setae; terminal chain divided into 10 segments. Scape length-to-width ratio 1.90 (Figure 25B).

Mandibles typical, apex with four distal teeth (Figure 26A).

Maxillary palp I with one processus basalis and one inner protuberance (Figure 26D,G). Maxillary palp articles without short brush-like setae ventrally. Ratio of ultimate article to penultimate article length 0.62. Dorsal surface of maxillary palp articles V–VII with transparent spines (Figure 26D,E); numbers given in Table 2.

Labial palp third segment slightly swollen apically, with sensory cones; numbers given in Table 2. Labial palp without long hair-like setae (Figure 26B,C).

**Thorax.** Thorax normal. Foreleg without sensory field or coxal styli; mid- and hind legs with coxal styli. Ventral surface of legs with long bristles, without needle-shaped setae. Pretarsal claw structure normal (Figure 26H–M).

**Abdomen.** Abdominal coxites I, VI, and VII with one pair of retractile vesicles; coxites II–V with two pairs of retractile vesicles (Figure 27A–G); inner lobes of female coxite VII fused and posteriorly extended (Figure 27G). Ratios of styli length to coxites length on abdominal segment V, styli length to supporting spines length, and basal width to length of sternites given in Table 5. Coxite IX medially with 4–5+4–5 transparent macrochaetae (Figure 27I). Caudal filament laterally and cerci medially with deciduous long hairs and supporting spines (Figure 27J).

Ovipositor of primary type, slightly shorter than styli (including supporting spines) of coxite IX (Figure 27H,I). Number of segments of anterior gonapophyses and posterior gonapophyses given in Table 6.

**Differential Diagnosis.** *Pedetontus* (*Verhoeffilis*) *shenzhenensis* sp. n. is morphologically closest to *P.* (*V.*) *bianchii* Silvestri, 1936, from Hong Kong. The abdominal segment IX provides a key distinction: in *P.* (*V.*) *shenzhengensis* sp. n., the styli (including supporting spines) of coxite IX are longer than coxite IX itself, while the posterior gonapophyses are slightly shorter than these styli. Conversely, in *P.* (*V.*) *bianchii*, the styli (including supporting spines) of coxite IX are shorter than coxite IX, and the posterior gonapophyses exceed their length. Additionally, the scape of *P.* (*V.*) *shenzhenensis* sp. n. is slender than that of *P.* (*V.*) *bianchii*, with length-to-width ratios of 1.90 and 1.42, respectively.

**Etymology.** The species is named after its type locality, Shenzhen City. The Chinese name for *P.* (*V.*) *shenzhenensis* sp. n. is 深圳跳蛃.

**Distribution.** Guangdong Province, China.

**Figure 28:**
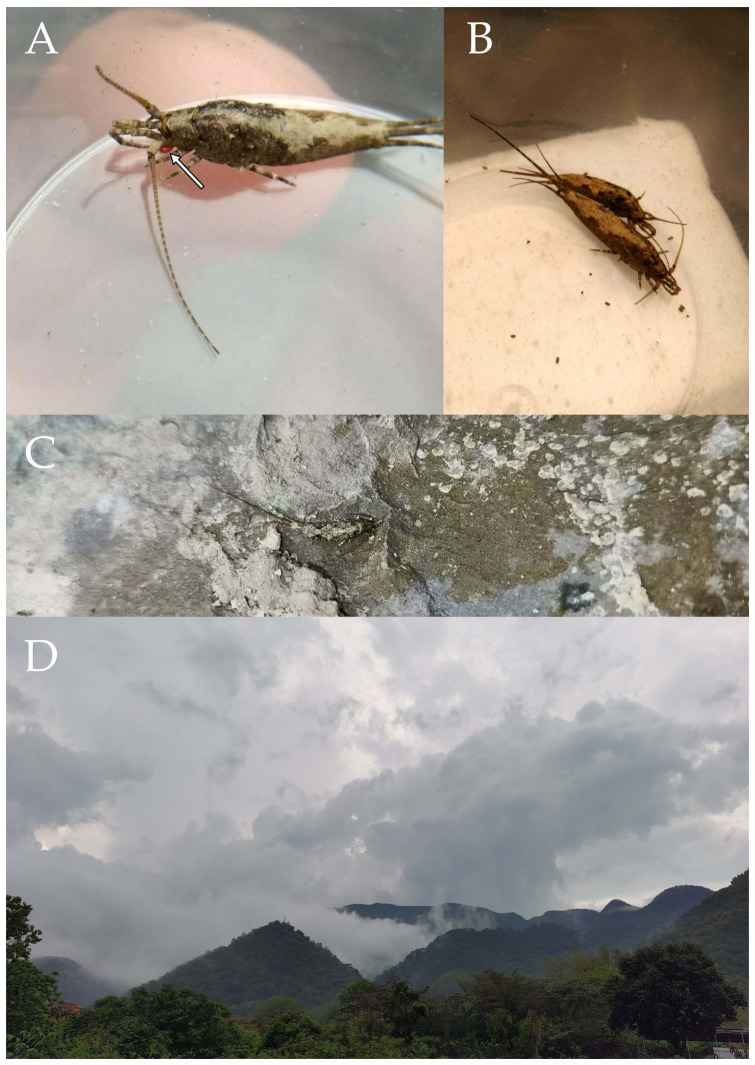
*Pedetontus* (*Verhoeffilis*) *xanthospilus* Shen, Yang, Ji & Zhang sp. n. (**A**) The captured *P.* (*V.*) *xanthospilus* sp. n., arrow indicates a mite. (**B**) Ditto. (**C**) In situ. (**D**) Habitat.

**Figure 29:**
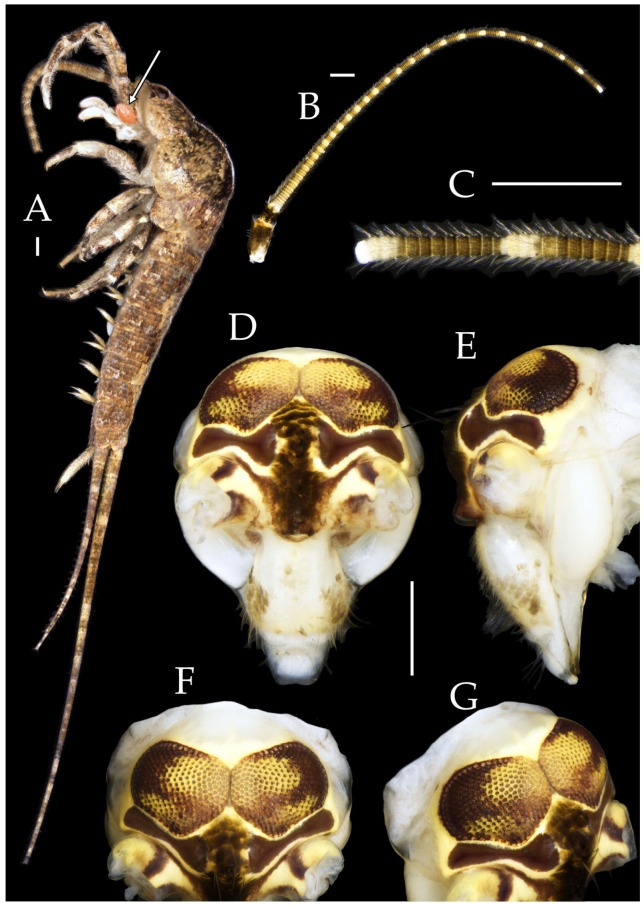
*Pedetontus* (*Verhoeffilis*) *xanthospilus* Shen, Yang, Ji & Zhang sp. n., holotype, male. (**A**) Habitus, lateral view, arrow indicates a mite attached to the maxilla. (**B**) Antenna. (**C**) Terminal chain of flagellum. (**D**) Head, frontal view. (**E**) Ditto, lateral view. (**F**,**G**) Compound eyes and paired ocelli. Scale bars: 500 μm.

**Figure 30:**
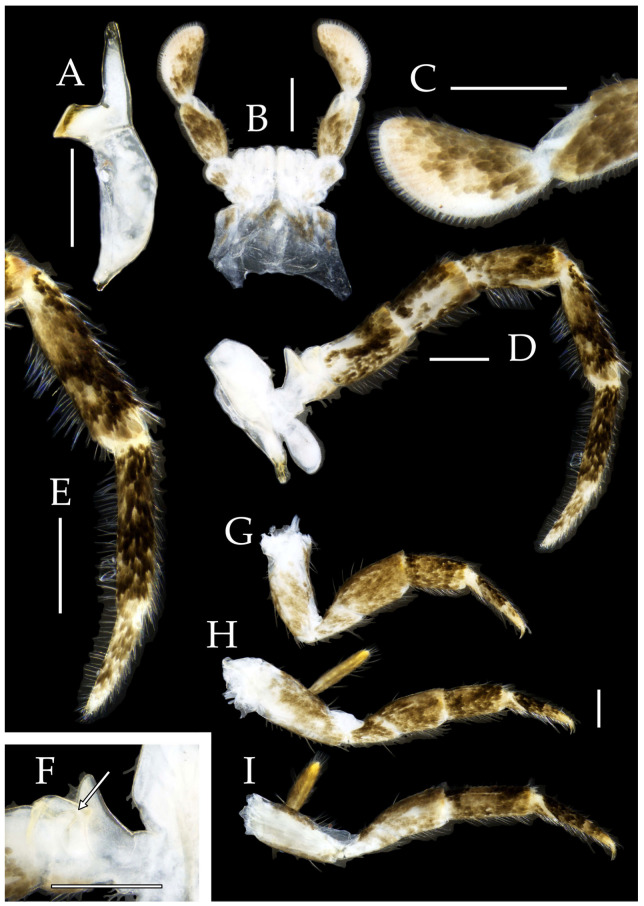
*Pedetontus* (*Verhoeffilis*) *xanthospilus* Shen, Yang, Ji & Zhang sp. n., holotype, male. (**A**) Mandible. (**B**) Labium. (**C**) Labial palp. (**D**) Maxilla. (**E**) Maxillary palp articles V–VII. (**F**) Maxillary inner protuberance, as indicates by the arrow. (**G**–**I**) Foreleg, midleg, and hindleg. Scale bars: 500 μm.

**Figure 31:**
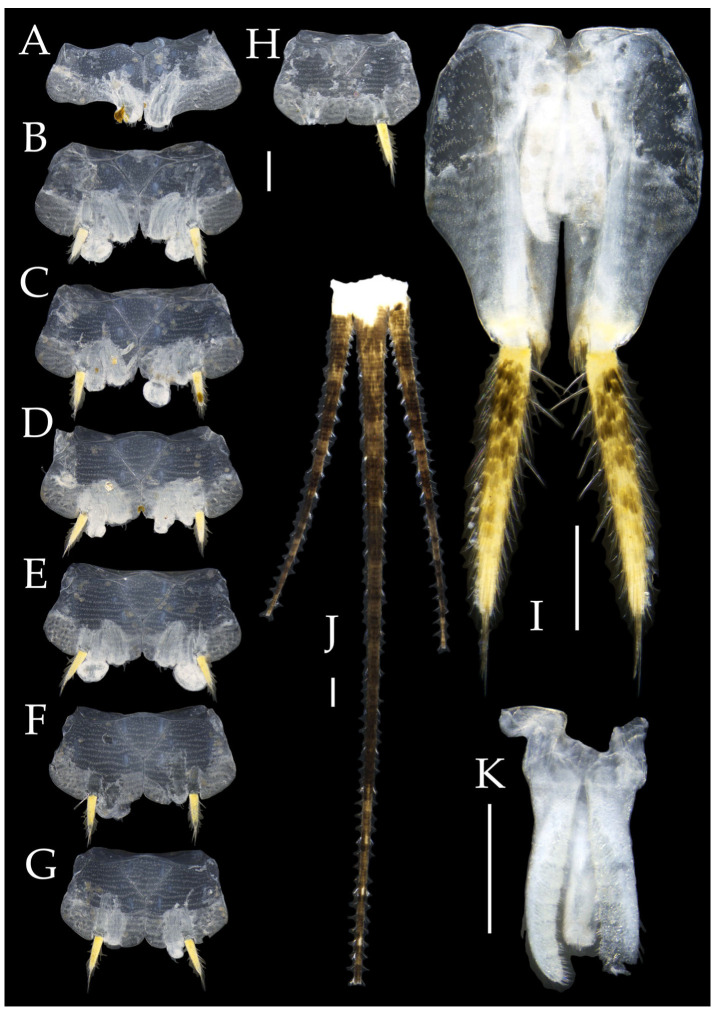
*Pedetontus* (*Verhoeffilis*) *xanthospilus* Shen, Yang, Ji & Zhang sp. n., holotype, male. (**A–I**) Abdominal sternites and coxites I–IX. (**J**) Caudal filament and cerci. (**K**) Penis and paramere. Scale bars: 500 μm.

**Figure 32:**
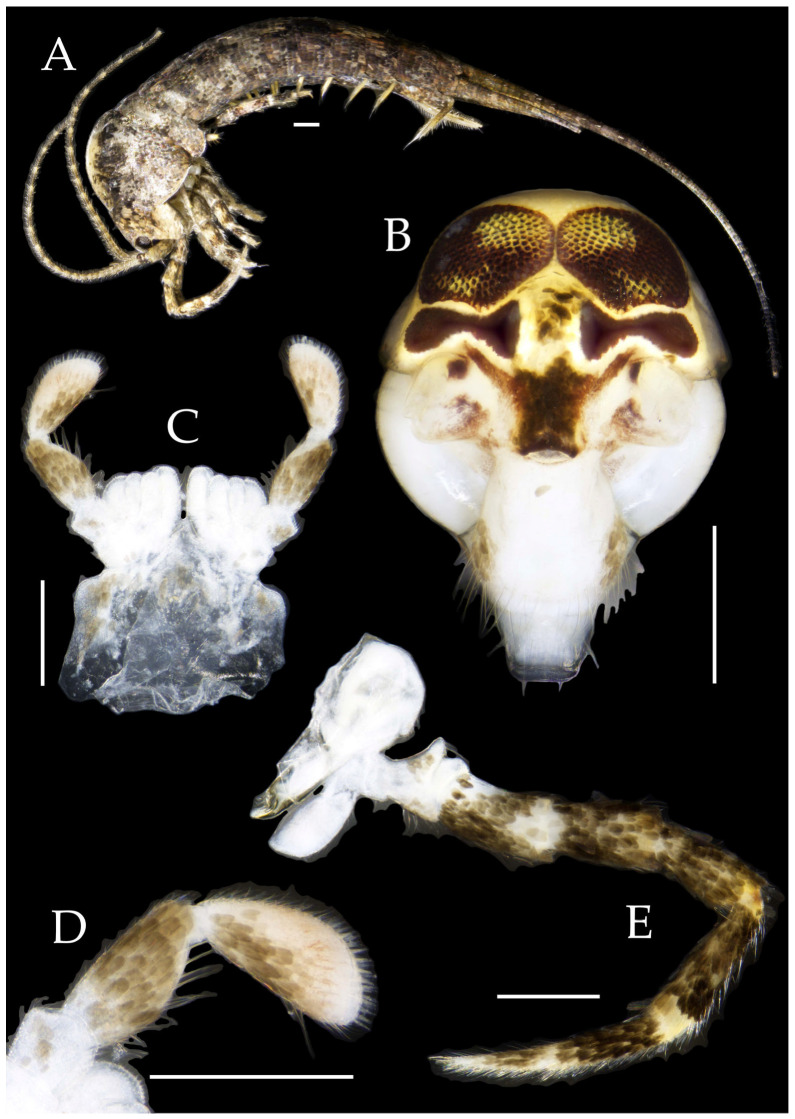
*Pedetontus* (*Verhoeffilis*) *xanthospilus* Shen, Yang, Ji & Zhang sp. n., paratype, female. (**A**) Habitus, lateral view. (**B**) Head, frontal view. (**C**) Labium. (**D**) Labial palp. (**E**) Maxilla. Scale bars: 500 μm.

**Figure 33:**
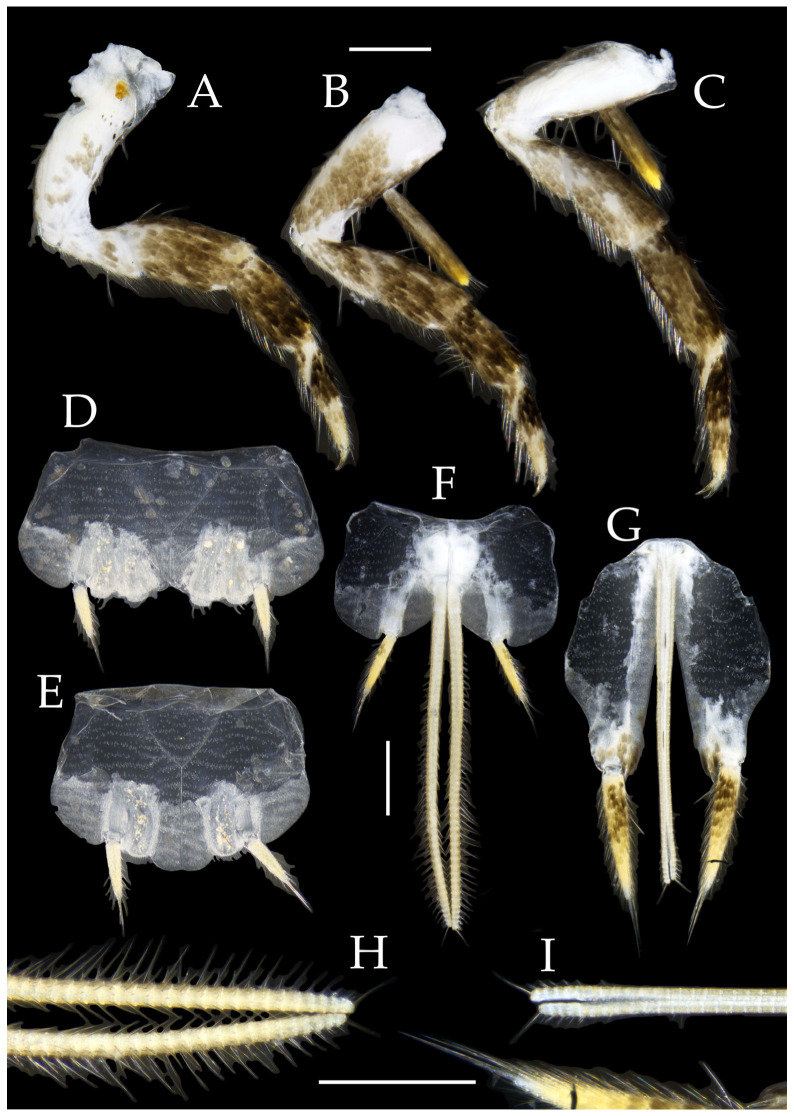
*Pedetontus* (*Verhoeffilis*) *xanthospilus* Shen, Yang, Ji & Zhang sp. n., paratype, female. (**A**–**C**) Foreleg, midleg, and hindleg. (**D–G**) Abdominal sternites and coxites V, VII–IX. (**H**) Apex of anterior gonapophyses. (**I**) Apex of posterior gonapophyses. Scale bars: 500 μm.

***Pedetontus* (*Verhoeffilis*) *xanthospilus* Shen, Yang, Ji & Zhang sp. n.** (Figure 28, Figure 29, Figure 30, Figure 31, Figure 32 and Figure 33)

Zoobank: urn:lsid:zoobank.org:act:8C07BFC0-2BB3-4053-A2AB-23B08DA13180

**Type Specimens and Type Locality.** Holotype, 1♂ (in 70% ethanol), paratypes: 11♂7♀ (in 70% ethanol), China, Guangdong Province, Zhaoqing City, Duanzhou District, Beiling Mountain Forest Park (23°09′ N 112°30′ E), from rocks along a stream, 20.XI.2024–3.I.2025, collected by Han-sheng Ou. All type specimens deposited in the Animal Herbarium, Zhejiang Normal University, Jinhua, China.

**Description.** Male body length 10.2 mm, female 9.2 mm. Body covered with dark brown scales; nota with a pale longitudinal stripe extending from the pronotum to the base of the caudal filament, terminating in two large and one small diamond-shaped markings. Antennae slightly shorter than body length; caudal filament slightly longer than body length; cerci about half as long as body length (Figure 28A–C, Figure 29A and Figure 32A).

**Head.** Compound eyes smooth, dark brown, with a large bright yellow blotch internally and a smaller one inferiorly (Figure 29D–G and Figure 32B); ratios of eye length to width and contact line length to eye length given in Table 1. Ocelli dark brown, dumbbell-shaped. Ratios of ocellus length to width and distance between inner margins of ocelli to combined width of eyes given in Table 1. Vertex without markings or setae. Frons convex, with long transparent bristles, scaled. Genae without markings or setae. Clypeus and labrum with transparent setae (Figure 29D–G and Figure 32B).

Antennae scaled except on flagellum; flagellum with distinct pale annulations, bearing well-developed annulate setae; terminal chain divided into 11–12 segments. Scape length-to-width ratio 1.74–1.91 (Figure 29B).

Mandibles typical, apex with four distal teeth (Figure 30A).

Maxillary palp I with one processus basalis and one inner protuberance (Figure 30D,F and Figure 32E). Maxillary palp articles II–VII with brush-like setae ventrally, setae on articles IV–V longer than others. Ratio of ultimate article to penultimate article length 0.45–0.52. Dorsal surface of maxillary palp articles V–VII with transparent spines (Figure 30D and Figure 32E); numbers given in Table 2.

Male labial palp third segment modified, laterally expanded, apex with sensory cones; numbers given in Table 2; ultimate article of female almost unswollen, clavate. Labial palp without long hair-like setae (Figure 30B,C and Figure 32D).

**Thorax.** Thorax normal. Foreleg without sensory field or coxal styli; mid- and hind legs with coxal styli, apex of coxal styli bright yellow. Ventral surface of legs with long bristles, without needle-shaped setae. Pretarsal claw structure normal (Figure 30G–I and Figure 33A–C).

**Abdomen.** Abdominal coxites I, VI, and VII with one pair of retractile vesicles; coxites II–V with two pairs of retractile vesicles; coxites I–IX each with a pair of yellow styli (Figure 31A–I and Figure 33D–G); inner lobes of female coxite VII fused and posteriorly extended (Figure 33E). Ratios of styli length to coxites length on abdominal segment V, styli length to supporting spines length, and basal width to length of sternites given in Table 5. Coxite IX medially with 4–5+4–5 transparent macrochaetae (Figure 31I and Figure 33G). Caudal filament laterally and cerci medially with deciduous long hairs and supporting spines (Figure 31J).

Coxite VIII without parameres. Coxite IX with penis and parameres (Figure 31I,K). Penis opening apically, shorter than parameres; parameres 1+7 segmented (Figure 31K). Ovipositor of primary type, not exceeding styli (including supporting spines) of coxite IX (Figure 33F,G). Number of segments of anterior gonapophyses and posterior gonapophyses given in Table 6.

**Differential Diagnosis.** See corresponding section of *Pedetontus* (*Verhoeffilis*) *jinxiuensis* sp. n.

**Etymology.** The specific epithet “xanthospilus” is a Latinized adjective derived from Greek xanthos (ξανθός), meaning “yellow”, and Greek spilos (σπίλος), meaning “blotch” or “stain”. It refers to the species’ conspicuous yellow blotches on the compound eyes, a defining diagnostic trait that distinguishes this taxon from congeners. The Chinese name for *P.* (*V.*) *xanthospilus* sp. n. is 黄斑跳蛃.

**Distribution.** Guangdong Province, China.

### 3.4. Phylogenetic Tree of Machilidae Based on COX1

**Figure 34:**
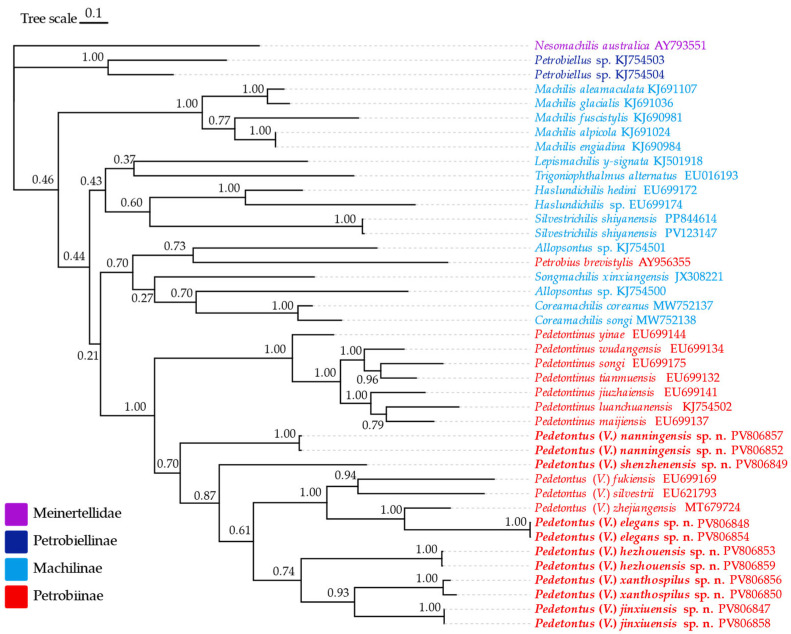
Bayesian inference phylogenetic tree based on a tandem dataset of 41 *COX1* genes. Posterior probabilities are shown at the nodes.

Based on the phylogenetic tree (Figure 34), Machilinae is a paraphyletic group. Also, the monophyly of Petrobiinae is refuted by the inclusion of *Petrobius brevistylis* within Machilinae, but the monophyly of the *Pedetontus* group is supported (*Pedetontus*+*Pedetontinus*). Within the genus *Pedetontus*, *P.* (*V.*) *nanningensis* sp. n. occupies the basal position, *P.* (*V.*) *elegans* sp. n. and *P.* (*V.*) *zhejiangensis* form a sister clade, while *P.* (*V.*) *xanthospilus* sp. n. and *P.* (*V.*) *jinxiuensis* sp. n. constitute another sister clade.

## 4. Discussion

Based on morphological observations and *COX1* gene analysis, this study reports six new species of the genus *Pedetontus* from southern China: *P.* (*V.*) *elegans* sp. n. from Zhejiang Province; *P.* (*V.*) *shenzhenensis* sp. n. and *P.* (*V.*) *xanthospilus* sp. n. from Guangdong Province; and *P.* (*V.*) *nanningensis* sp. n., *P.* (*V.*) *hezhouensis* sp. n., and *P.* (*V.*) *jinxiuensis* sp. n. from Guangxi Zhuang Autonomous Region. All these species belong to the subgenus *Verhoeffilis*, which is characterized by the presence of two pairs of retractile vesicles restricted to coxites II–V and posterior angles of abdominal sternites ≤90°. *P.* (*V.*) *elegans* sp. n. and *P.* (*V.*) *zhejiangensis* form a sister clade; both species originate from Zhejiang Province and share some morphological similarities, but they can be distinguished by a few characteristics. The compound eyes of *P.* (*V.*) *zhejiangensis* are reddish-brown, while those of *P. elegans* sp. n. are yellowish-green. *P.* (*V.*) *zhejiangensis* lacks stout setae on the frons, whereas *P.* (*V.*) *elegans* sp. n. possesses well-developed stout setae. The male labial palps of *P.* (*V.*) *zhejiangensis* bear a few long filamentous setae laterally, which are absent in *P.* (*V.*) *elegans* sp. n. The ultimate segment of the maxillary palps is distinctly shorter than the penultimate segment in *P.* (*V.*) *zhejiangensis*, whereas in *P.* (*V.*) *elegans* sp. n., the ultimate and penultimate segments are equal in length. The parameres of *P.* (*V.*) *zhejiangensis* exhibit 1+8 segmentation, and the penis is shorter than the parameres; in contrast, *P.* (*V.*) *elegans* sp. n. has 1+7 segmented parameres, and the penis is longer than the parameres.

The subgenus *Verhoeffilis* is believed to have originated in the southeastern Palaearctic Realm. Its northeastward migration led to the emergence of the American *s. stricto* subgenus, whereas its southward dispersal resulted in the current Indo-Malayan *Pedetontus* species [6]. Southern China is situated at the transitional zone between the Palaearctic and Oriental Realms. Among the new species described, *P.* (*V.*) *elegans* sp. n. (Zhejiang) exhibits morphological traits most consistent with the Palaearctic fauna: compound eyes longer than wide; ventral surfaces of thoracic legs bearing subspine-form setae. In contrast, the remaining five species (from Guangdong and Guangxi) possess compound eyes that are wider than long and lack subspine-form setae on thoracic legs. Phylogenetic analysis indicates that *P.* (*V.*) *elegans* sp. n. is closely related to the Palaearctic species *P. silvestrii*.

*P.* (*V.*) *xanthospilus* sp. n. and *P.* (*V.*) *jinxiuensis* sp. n. form sister clades on the phylogenetic tree and exhibit morphological similarities. However, the genetic distance based on *COX1* sequences and differences in the labial palps provide evidence that they represent distinct species. These observations suggest the subgenus is undergoing a process of divergence.

*Petrobius brevistylis* is clustered within the *Allopsontus* group—an unexpected placement that highlights its taxonomic distinctness. This is consistent with its possession of non-segmented parameres and a penis that is significantly longer than the coxite IX, in contrast to most Petrobiinae species, which typically exhibit segmented parameres and a penis shorter than the coxite IX [55]. The genus *Allopsontus* appears to be non-monophyletic according to the phylogenetic tree, potentially due to the presence of seven morphologically distinct subgenera distributed across Northwest China, Central Asia, Mongolia, and the southern Himalayas. Key morphological differences include variations in ocellar shape/position, the number of coxal vesicles, and penis morphology [56], suggesting a lack of monophyly that warrants further investigation through molecular and detailed morphological study.

## Figures and Tables

**Table 1:** Ratio of compound eyes and ocelli of *Pedetontus* new species.

Ratio	Eye Length to Width	Eye Contact Line Length to Eye Length	Ocellus Length to Width	Distance Between Inner Margins of Ocelli to Combined Width of Eyes
***P.*** **(*V.*)** ***elegans*** **sp. n.**	1.01–1.09	0.59–0.71	0.30–0.33	0.19–0.22
***P.*** **(*V.*) *hezhouensis*** **sp. n.**	0.79–0.84	0.32–0.39	0.35–0.43	0.18
***P.*** **(*V.*) *jinxiuensis*** **sp. n.**	0.81–0.85	0.20–0.25	0.33–0.36	0.15–0.16
***P.*** **(*V.*)** ***nanningensis*** **sp. n.**	0.82–0.84	0.31–0.37	0.38–0.40	0.10–0.15
***P.*** **(*V.*) *shenzhenensis*** **sp. n.**	0.84	0.41	0.40	0.19
***P.*** **(*V.*) *xanthospilus*** **sp. n.**	0.83–0.84	0.27–0.28	0.36	0.21–0.22

**Table 2:** Numbers of transparent spines on dorsal surfaces of maxillary palps and sensory cones on apex of labial palp III of *Pedetontus* new species.

Species		Number of Dorsal Transparent Spines on Maxillary Palps			Sensory Cones on Apex of Labial Palp III
		V	VI	VII	
***P.*** **(*V.*)** ***elegans*** **sp. n.**	Male	1–4	6–14	7–14	25–31
	Female	2–3	10–16	10–14	23–26
***P.*** **(*V.*) *hezhouensis*** **sp. n.**	Male	2	7	6–7	50
	Female	4–5	11–15	11–13	35–39
***P.*** **(*V.*) *jinxiuensis*** **sp. n.**	Male	0–1	11–12	8–9	0–43
	Female	4	12	15	0
***P.*** **(*V.*)** ***nanningensis*** **sp. n.**	Male	3	12	9–11	46–50
	Female	6–7	16	15–16	36–40
***P.*** **(*V.*) *shenzhenensis*** **sp. n.**	Male	N/A	N/A	N/A	N/A
	Female	5–6	16–17	13	36–45
***P.*** **(*V.*) *xanthospilus*** **sp. n.**	Male	0–2	10	12–14	55–58
	Female	2–3	12–14	11–12	36–38

**Table 3:** Number of needle-shaped setae on ventral surfaces of legs of *Pedetontus* (*Verhoeffilis*) *elegans* sp. n.

Legs			Number of Needle-Shaped Setae on Ventral Surfaces
**Foreleg**	Femur		0
	Tibia		0
	Tarsi	Tarsomere I	2
		Tarsomere II	5
		Tarsomere III	0
**Midleg**	Femur		0
	Tibia		0
	Tarsi	Tarsomere I	3
		Tarsomere II	6
		Tarsomere III	1
**Hindleg**	Femur		0
	Tibia		4
	Tarsi	Tarsomere I	3
		Tarsomere II	10
		Tarsomere III	

**Table 4:** Angles of posterior angles (°) of abdominal sternites of *Pedetontus* new species.

Species	Urosternite I	Urosternite II	Urosternite III	Urosternite IV	Urosternite V	Urosternite VI	Urosternite VII	Urosternite VIII (Male)
***P. elegans* (*V.*) sp. n.**	129–138	78–92	88–93	92–94	86–95	74–92	99–113	123
***P. hezhouensis* (*V.*) sp. n.**	129–131	78–87	78–83	82–83	81–84	91–96	89–100	91
***P. jinxiuensis* (*V.*) sp. n.**	129–141	68–76	75–77	79–82	75–84	79–95	79–97	95
***P. nanningensis* (*V.*) sp. n.**	123–129	67–88	68–82	78–86	83–88	79–88	80–101	84
***P. shenzhenensis* (*V.*) sp. n.**	140	80	73	77	77	77	81	N/A
***P. xanthospilus* (*V.*) sp. n.**	123–129	76–83	73–80	78–80	84–86	82–83	83–89	80

**Table 5:** Ratios of styli length to coxites length on abdominal segment V, styli length to supporting spines length, and basal width to length of sternite.

Ratio	Styli (Without Supporting Spines) Length to Coxites Length of Abdominal Segment V	Styli (Without Supporting Spines) Length to Supporting Spines Length of Abdominal Segment V	Basal Width to Length of Sternite of Abdominal Segment V
***P.* (*V.*) *elegans* sp. n.**	0.58–0.64	0.40–0.43	1.33–1.34
***P.* (*V.*)** ***hezhouensis* sp. n.**	0.42–0.48	0.53–0.58	1.08–1.24
***P.* (*V.*)** ***jinxiuensis* sp. n.**	0.37–0.43	0.65–0.71	1.18–1.21
***P.*** **(*V.*) *nanningensis* sp. n.**	0.43	0.52	1.06–1.36
***P.*** **(*V.*)** ***shenzhenensis*** **sp. n.**	0.47	0.60	1.20
***P.* (*V.*)** ***xanthospilus* sp. n.**	0.38–0.41	0.55–0.68	1.19–1.25

**Table 6:** Number of segments of anterior gonapophyses and posterior gonapophyses of *Pedetontus* new species.

Species	Number of Segments of Anterior Gonapophyses	Number of Segments of Posterior Gonapophyses
***P.* (*V.*) *elegans* sp. n.**	56–59	58–63
***P.* (*V.*)** ***hezhouensis* sp. n.**	60–67	53–55
***P.* (*V.*)** ***jinxiuensis* sp. n.**	60	56–58
***P.*** **(*V.*) *nanningensis* sp. n.**	59	50–55
***P.*** **(*V.*)** ***shenzhenensis*** **sp. n.**	42–54	48–49
***P.* (*V.*)** ***xanthospilus* sp. n.**	50–51	49

## Data Availability

Supporting data for this study are available from the National Center for Biotechnology Information (https://www.ncbi.nlm.nih.gov) (accessed on 10 June 2025). For GenBank numbers, see Figure 34 and Table S1.

## Supplementary Materials

The following supporting information can be downloaded at https://www.mdpi.com/article/10.3390/insects16090916/s1, Table S1: Information about samples used in this study and their NCBI GenBank accession numbers.
