# Supplementary material for: Six New Species of Genus Pedetontus Silvestri, 1911 (Microcoryphia: Machilidae), from Southern China
Source: Insects. 2025 Sep 1;16(9):916. doi: 10.3390/insects16090916 (PMC12470564; doi:10.3390/insects16090916)
Supplement: Supplementary file 1 [file insects-16-00916-s001.zip › insects-3794494-supplementary.pdf]

Table S1. Information about samples used in this study and their NCBI GenBank accession numbers.

S/N	Species	Family	Accession No.	Reference
1	*Nesomachilis australica*	Meinertellidae	AY793551	[1]
2	*Allopsontus* sp.	Machilidae	KJ754500	[2]
3	*Allopsontus* sp.	Machilidae	KJ754501	[2]
4	*Coreamachilis coreanus*	Machilidae	MW752137	[3]
5	*Coreamachilis songi*	Machilidae	MW752138	[3]
6	*Haslundichilis hedini*	Machilidae	EU699172	[4]
7	*Haslundichilis* sp.	Machilidae	EU699174	[4]
8	*Lepismachilis* sp.	Machilidae	EU699162	[4]
9	*Lepismachilis y-signata*	Machilidae	KJ501918	[5]
10	*Machilis aleamaculata*	Machilidae	KJ691107	Direct submission
11	*Machilis alpicola*	Machilidae	KJ691024	Direct submission
12	*Machilis engiadina*	Machilidae	KJ690984	Direct submission
13	*Machilis fuscistylis*	Machilidae	KJ690981	Direct submission
14	*Machilis glacialis*	Machilidae	KJ691036	Direct submission
15	*Pedetontinus luanchuanensis*	Machilidae	KJ754502	[2]
16	*Pedetontinus tianmuensis*	Machilidae	EU699132	[4]
17	*Pedetontinus wudangensis*	Machilidae	EU699134	[4]
18	*Pedetontinus maijiensis*	Machilidae	EU699137	[4]
19	*Pedetontinus jiuzhaiensis*	Machilidae	EU699141	[4]
20	*Pedetontinus yinae*	Machilidae	EU699144	[4]
21	*Pedetontinus songi*	Machilidae	EU699175	[4]
22	*Pedetontus fukiensis*	Machilidae	EU699169	[4]
23	*Pedetontus silvestrii*	Machilidae	EU621793	[6]
24	*Pedetontus xanthospilus* sp. n. GDZQ0118-28	Machilidae	PV806850	This study
25	*Pedetontus xanthospilus* sp. n. GDZQ0401-39	Machilidae	PV806856	This study
26	*Pedetontus elegans* sp. n. JHPA0331-38	Machilidae	PV806854	This study
27	*Pedetontus jinxiuensis* sp. n. GXDYS0409-43	Machilidae	PV806858	This study
28	*Pedetontus hezhouensis* sp. n. GXHZ0227-36	Machilidae	PV806853	This study
29	*Pedetontus hezhouensis* sp. n. GXHZ0417-44	Machilidae	PV806859	This study
30	*Pedetontus jinxiuensis* sp. n. GXJX0703-1	Machilidae	PV806847	This study
31	*Pedetontus nanningensis* sp. n. GXNN0218-33	Machilidae	PV806852	This study
32	*Pedetontus nanningensis* sp. n. GXNN0409-42	Machilidae	PV806857	This study
33	*Pedetontus elegans* sp. n. JHPA1114-18	Machilidae	PV806848	This study
34	*Pedetontus shenzhenensis* sp. n. SZYTS1126-23	Machilidae	PV806849	This study
35	*Pedetontus zhejiangensis*	Machilidae	MT679724	[7]
36	*Petrobiellus* sp.	Machilidae	KJ754503	[2]
37	*Petrobiellus* sp.	Machilidae	KJ754504	[2]
38	*Petrobius brevistylis*	Machilidae	AY956355	[8]
39	*Silvestrichilis shiyanensis*	Machilidae	PP844614	Direct submission
40	*Silvestrichilis shiyanensis*	Machilidae	PV123147	Direct

				submission
41	*Songmachilis xinxiangensis*	Machilidae	JX308221	[9]
42	*Trigoniophthalmus alternatus*	Machilidae	EU016193	[10]

Reference

1. Cameron, S.L.; Miller, K.B.; D'Haese, C.A.; Whiting, M.F.; Barker, S.C. Mitochondrial Genome Data Alone Are Not Enough to Unambiguously Resolve the Relationships of Entognatha, Insecta and Crustacea Sensu Lato (Arthropoda). *Cladistics* **2004**, *20*, 534–557.
2. Ma, Y.; He, K.; Yu, P.P.; Yu, D.N.; Cheng, X.F.; Zhang, J.Y. The Complete Mitochondrial Genomes of Three Bristletails (Insecta: Archaeognatha): The Paraphyly of Machilidae and Insights into Archaeognathan Phylogeny. *PLoS One* **2015**, *10*, e0117669.
3. Guan, J.Y.; Shen, S.Q.; Zhang, Z.Y.; Xu, X.D.; Storey, K.B.; Yu, D.N.; Zhang, J.Y. Comparative Mitogenomes of Two *Coreamachilis* Species (Microcoryphia: Machilidae) along with Phylogenetic Analyses of Microcoryphia. *Insects* **2021**, *12*, 795.
4. Zhang, J.Y.; Zhou, K.Y. Descriptions of One New Genus and Six New Species of Machilidae (Insecta: Archaeognatha) from China: Morphological and Molecular Data. *Journal of Natural History* **2011**, *45*, 1131–1164, doi:10.1080/00222933.2011.552801.
5. Gassner, M.; Dejaco, T.; Schönswetter, P.; Marec, F.; Arthofer, W.; Schlick-Steiner, B.C.; Steiner, F.M. Extensive Variation in Chromosome Number and Genome Size in Sexual and Parthenogenetic Species of the Jumping-Bristletail Genus *Machilis* (Archaeognatha). *Ecology and Evolution* **2014**, *4*, 4093–4105.
6. Zhang, J.Y.; Song, D.X.; Zhou, K.Y. The Complete Mitochondrial Genome of the Bristletail *Pedetontus silvestrii* (Archaeognatha: Machilidae) and an Examination of Mitochondrial Gene Variability within Four Bristletails. *Annals of the Entomological Society of America* **2008**, *101*, 1131–1136, doi:10.1603/0013-8746-101.6.1131.
7. Shen, S.Q.; Cai, Y.Y.; Xu, K.K.; Chen, Q.P.; Cao, S.S.; Yu, D.N.; Zhang, J.Y. The Complete Mitochondrial Genome of *Pedetontus zhejiangensis* (Microcoryphia: Machilidae) and Its Phylogeny. *Mitochondrial DNA Part B* **2020**, *5*, 3143–3145.
8. Podsiadlowski, L. The Mitochondrial Genome of the Bristletail *Petrobius brevistylis* (Archaeognatha: Machilidae). *Insect Molecular Biology* **2006**, *15*, 253–258.
9. He, K.; Zhang, J.Y.; Deng, K.Z.; Chen, Z. The Complete Mitochondrial Genome of the Bristletail *Songmachilis xinxiangensis* (Archaeognatha: Machilidae). *Mitochondrial DNA* **2013**, *24*, 99–101.
10. Carapelli, A.; Liò, P.; Nardi, F.; Van der Wath, E.; Frati, F. Phylogenetic Analysis of Mitochondrial Protein Coding Genes Confirms the Reciprocal Paraphyly of Hexapoda and Crustacea. *BMC evolutionary biology* **2007**, *7*, S8.
